# Equations of Motion of Free-Floating Spacecraft-Manipulator Systems: An Engineer's Tutorial

**DOI:** 10.3389/frobt.2018.00041

**Published:** 2018-04-18

**Authors:** Markus Wilde, Stephen Kwok Choon, Alessio Grompone, Marcello Romano

**Affiliations:** ^1^Department of Mechanical and Aerospace Engineering, Florida Institute of Technology, Melbourne, FL, United States; ^2^Spacecraft Robotics Laboratory, Mechanical and Aerospace Engineering Department, Naval Postgraduate School, Monterey, CA, United States

**Keywords:** robot dynamics modeling, spacecraft manipulator dynamics, generalized Jacobian, generalized inertia matrix, Lagrangian equations of motion

## Abstract

The paper provides a step-by-step tutorial on the Generalized Jacobian Matrix (GJM) approach for modeling and simulation of spacecraft-manipulator systems. The General Jacobian Matrix approach describes the motion of the end-effector of an underactuated manipulator system solely by the manipulator joint rotations, with the attitude and position of the base-spacecraft resulting from the manipulator motion. The coupling of the manipulator motion with the base-spacecraft are thus expressed in a generalized inertia matrix and a GJM. The focus of the paper lies on the complete analytic derivation of the generalized equations of motion of a free-floating spacecraft-manipulator system. This includes symbolic analytic expressions for all inertia property matrices of the system, including their time derivatives and joint-angle derivatives, as well as an expression for the generalized Jacobian of a generic point on any link of the spacecraft-manipulator system. The kinematics structure of the spacecraft-manipulator system is described both in terms of direction-cosine matrices and unit quaternions. An additional important contribution of this paper is to propose a new and more detailed definition for the modes of maneuvering of a spacecraft-manipulator. In particular, the two commonly used categories free-flying and free-floating are expanded by the introduction of five categories, namely floating, rotation-floating, rotation-flying, translation-flying, and flying. A fully-symbolic and a partially-symbolic option for the implementation of a numerical simulation model based on the proposed analytic approach are introduced and exemplary simulation results for a planar four-link spacecraft-manipulator system and a spatial six-link spacecraft manipulator system are presented.

## Introduction

Small spacecraft equipped with robotic manipulators will be the backbone of any robotic on-orbit servicing, asteroid mining, or active space debris removal missions. Examples for such missions are discussed in more detail by Yoshida (2003), Shoemaker and Wright (2004), Reintsema et al. (2010), Barnhart et al. (2013). The use of spacecraft-mounted robotic manipulator systems for the assembly and maintenance of spacecraft has a long and successful history with the Space Shuttle and International Space Station programs. The Space Shuttle orbiters were equipped with the Shuttle Remote Manipulator System, colloquially known as *Canadarm* (Sallaberger, 1997). The SRMS was successfully used to capture the Hubble space telescope and other satellites during servicing missions, to position astronauts during extra-vehicular activities, and to assemble and resupply the ISS (Goodman, 2006; Hale et al., 2011). The ISS is currently equipped with two manipulator systems, the Space Station Remote Manipulator System (Stieber et al., 1997), also known as *Canadarm 2*, and the Japanese Experiment Module Remote Manipulator System (Sato and Wakabayashi, 2001). The Space Station Remote Manipulator System is used to capture and berth H-II Transfer Vehicle (HTV), *Dragon*, and *Cygnus* vehicles, and to position supplies and astronauts (Dreyer, 2009; Bain, 2010; Ueda et al., 2010). With the Special Purpose Dexterous Manipulator (also called *Dextre*) as end-effector, the Space Station Remote Manipulator System is capable of fine manipulation (Coleshill et al., 2009). This capability is, for example, used in the NASA Robotic Refueling Mission, which demonstrated the feasibility of accessing and refueling a typical satellite fuel port (Cepollina, 2013). The Japanese Experiment Module Remote Manipulator System is mostly used to service the Japanese Experiment Module *Kibo* and is also equipped with a dexterous end-effector. The manipulator suite of the ISS is planned to be completed with the installation of the European Robotic Arm on the Russian module (Patten et al., 2002). The use of robotic manipulator arms mounted on small servicing spacecraft to capture and manipulate a client satellite has successfully been demonstrated with the Engineering Test Satellite VII (ETS-VII) and Orbital Express missions (Yoshida, 2003; Kennedy, 2008). The upcoming on-orbit servicing demonstration missions Restore-L and Robotic Servicing of Geostationary Satellites will also feature robotic manipulators as core component of the capture and servicing system (Reed et al., 2016; Roesler et al., 2017).

Operating a robotic manipulator on a spacecraft results in a complex system overlapping the disciplines of robotics and aerospace engineering. Since the dynamics between the manipulator and the base-spacecraft are coupled, the system requires an integrated control system to meet the capture or manipulation goals and ensure mission success. Moreover, the complex dynamics of the spacecraft-manipulator system must be accounted for in the maneuver planning and in its overall maneuver timeline. The effects of manipulator operations on the orientation and position of the Shuttle orbiter and the ISS have been small and could be managed through operational procedures (Sargent, 1984). For fast-moving manipulators mounted on small base-spacecraft, the position and orientation disturbances due to manipulator motion become critical, as demonstrated on ETS-VII and Orbital Express (Oda, 2000; Kennedy, 2008). Hence, the engineers developing the spacecraft control system, specifying the sensor systems to be used, developing the communications system, and developing the operations plan must have an understanding of the complex dynamics arising from the multi-body system. However, multi-body dynamics and modeling of robotic systems are not part of a typical undergraduate aerospace engineering curriculum and are rarely covered in aerospace engineering graduate programs. Therefore, aerospace engineers must resort to the academic literature to fill this knowledge gap. Available literature focusses on the application and performance analysis of various approaches to describing the coupled dynamics of a spacecraft-manipulator system (Moosavian and Papadopulos, 2007; Flores-Abad et al., 2014), but does not provide a complete description of the modeling approach that would allow an aerospace engineer to access the topic without consulting a combination of research publications and textbooks.

There are two common methods for modeling the dynamics and deriving the equations of motion of multibody systems: the recursive Newton-Euler method and the Lagrangian method. A general description of the Newton-Euler method is given in Siciliano et al. (2010). Stoneking (2007) provides a detailed presentation of the use of Newton-Euler dynamics to develop the equations of motion for a multibody spacecraft. Applications in the field of spacecraft-manipulator dynamics are shown in Longman et al. (1987) and Mukherjee and Nakamura (1992). In the Newton-Euler method, the equations of motion of the multibody system are computed from the equilibria of forces and torques acting on each link of the system. From this, a recursive algorithm can be developed. In the forward recursion through the structure of the multibody system, the linear and angular link accelerations and velocities are computed. The forces and moments acting on the links are then computed in the backward recursion. To develop the equations of motion of a system of flexible links, the Direct Path Method was developed (Ho, 1977; Hughes, 1979). In the Direct Path Method, the point of reference of the equations of motion is moved from the system center-of-mass to a fixed point in one of the bodies, which is typically selected to be the center-of-mass of the base spacecraft. The structure of the system is then described following the most direct path through the links. The torques acting on the links are taken about the joints instead of the link centers-of-mass, thus eliminating constraint forces and torques between the links.

The Lagrangian method develops the equations of motion of a multibody system from its kinetic and potential energies, using a set of generalized coordinates describing the positions of the links (Siciliano et al., 2010). Following Siciliano et al. (2010), the Lagrangian method is advantageous in it being systematic and easily comprehensible and in providing the equations of motion in a compact analytical form facilitating control systems design. The fundamental advantage of the Newton-Euler approach is its computational efficiency as a recursive algorithm. The Lagrangian method is thus used to explain the development of the equations of motion in the present paper, due to its systematic nature.

When controlling the motion of the spacecraft-manipulator system, the dynamic coupling of the base spacecraft and the manipulator becomes a concern. Since the base spacecraft in one of the five maneuvering modes described in section Detailed Classification of Spacecraft-Manipulator System Maneuvering, and thus not fixed in space, any motion of the manipulator will cause a rotation and translation of the base spacecraft. A comprehensive overview of methods to account for the dynamic coupling in controlling the position and orientation of both the end-effector of the manipulator and the base-spacecraft is provided in Flores-Abad et al. (2014). Three of these methods shall be highlighted here: The Virtual Manipulator (VM) approach, the Dynamically Equivalent Manipulator (DEM) approach, and the Generalized Jacobian Matrix (GJM) approach. The VM approach replaces the physical spacecraft-manipulator system with a dynamically consistent VM system (Vafa and Dubowsky, 1987). The base of the VM is a spherical joint located at the center-of-mass of the physical spacecraft-manipulator system. The orientation of this joint equals the orientation of the base-spacecraft in inertial space. Since, in the absence of external forces, the system center-of-mass remains stationary, the complex free-floating system is replaced by a dynamically consistent fixed-base system. The VM is a purely kinematic computational model in the sense that it is a massless kinematic chain with constant dimensions calculated from the geometry and mass properties of the spacecraft-manipulator system. It is typically used for workspace analysis for floating spacecraft-manipulator systems (Vafa and Dubowsky, 1987; Dubowsky and Papadopoulos, 1993). In particular, the VM cannot be represented by a physical manipulator. However, this can be achieved by the DEM approach (Liang et al., 1998). The base joint of the DEM is also a spherical joint at the system center-of-mass, and the DEM is geometrically identical to the VM for the same spacecraft-manipulator system. Differing from the VM, the DEM links have masses and moments-of-inertia matrices that are calculated from the mass distributions of the real system. Therefore, the DEM computation model can be reproduced in a real mechanical system, and thus be used in experimental systems.

The Generalized Jacobian approach was originally proposed by Yoshida and Umetani (1993), expanding the dynamic analysis previously introduced by Dubowsky and Papadopoulos (1993) and in the dissertation of Papadopoulos (1990). The Generalized Jacobian approach was used successfully in running simulations and developing control algorithms for the ETS-VII demonstrator mission (Yoshida, 2003). The approach was also further generalized to serve as a description for any under-actuated manipulator system (Yoshida and Nenchev, 1998). The Generalized Jacobian method formed the basis for the development of non-holonomic path planning algorithms (Nakamura and Mukherjee, 1989; Xu et al., 2008), target capture algorithms (Yoshida and Nakanishi, 2003), spacecraft-manipulator control strategies (Marchesi, 1997; Caccavale and Siciliano, 2001; Aghili, 2009), hardware-in-the-loop simulation of space robotic systems (Wei et al., 2006), Reaction Null-Space Control algorithms (Nenchev et al., 1999) and contact dynamics models (Nenchev and Yoshida, 1999). The method is advantageous for a symbolic analytic description of the dynamics of a complex spacecraft-manipulator system, which is important for the synthesis of guidance and control laws, as well as to guide the selection of proximity operations sensor systems and the design of pointing mechanisms for antennas, solar arrays, etc.

Notwithstanding the widespread use of the GJM approach, a complete description for the computation of all inertial parameters of the spacecraft-manipulator system is missing in the literature for the general case of a spatial *N*-link manipulator mounted on a six degrees-of-freedom (DOF) base-spacecraft. However, this is critical for its use in the design of the base spacecraft and the overall rendezvous and capture system and mission. This paper aims at filling this significant gap by presenting a complete symbolic, step-by-step derivation of the coupled equations of motion of a spacecraft-manipulator system following the GJM approach, including the time and joint angle derivatives of the generalized inertia matrix. The tutorial presented here uses the Lagrangian method for a single manipulator mounted on a base-spacecraft, under the assumption of zero linear and angular momenta, which makes it applicable to the description of the majority of current spacecraft-manipulator systems. The kinematics of the spacecraft-manipulator system are described both using direction cosine matrices and quaternions. For the extension of this approach to multi-manipulator systems, the reader is referred to Moosavian and Papadopoulos (2004), while the equations of motion of a spacecraft-manipulator system with non-zero angular momentum is covered in Nanos and Papadopoulos (2011). This complete discussion of the GJM approach enables the computation of symbolic expressions of the spacecraft-manipulator system equations of motion, which can then be used for the formulation of guidance and control laws in addition to numerical simulations.

A second important contribution of the paper is the introduction of a new, more accurate and detailed categorization for spacecraft-manipulator control modes. In particular, the two commonly used categories (free-flying and free-floating) are expanded by the introduction of five categories (namely floating, rotation-floating, rotation-flying, translation-flying, and flying).

A third contribution of the paper is the definition of the generalized Jacobian for an arbitrary point of the spacecraft-manipulator system, which is useful for guidance and control, obstacle avoidance, and collision detection and modeling.

To the authors' knowledge, this is the first time that the Generalized Jacobian approach is derived in completeness, drawing from multiple references in literature and the authors' own research. The paper thus forms a valuable resource for aerospace engineers to understand and model the complex dynamics of a robotic manipulator operated on a spacecraft.

The paper is organized as follows. Section Detailed Classification of Spacecraft-Manipulator System Maneuvering provides a detailed classification of the maneuvering modes of a spacecraft-manipulator system. Section Kinematics of a Spacecraft-Manipulator System develops expressions for the kinematics of a spacecraft-manipulator system based on a customized version of Denavit-Hartenberg parameters. Using these expressions, the dynamics of a floating spacecraft-manipulator system are described in section Dynamics of a Floating Spacecraft-Manipulator System. The paper proceeds to develop the generalized form of the equations of motion of a floating spacecraft-manipulator system from section Generalized Form of the Equations of Motion for a Floating Spacecraft-Manipulator System, followed by the generalized form of the generic Jacobians of a spacecraft-manipulator system in section Generalized Jacobian. Section Implementation of a Simulation Model for a Floating Spacecraft-Manipulator System then discusses the implementation options for a computer simulation model based on the generalized approach. The results of sample simulations are reported in section Numerical Simulations. Finally, section Conclusion provides concluding remarks.

## Detailed classification of spacecraft-manipulator system maneuvering

The spacecraft-manipulator system can maneuver in different modes, typically designated by the terms *free-floating* and *free-flying* (Umetani and Yoshida, 1989; Dubowsky and Papadopoulos, 1993). To arrive at a more detailed and complete classification of spacecraft maneuvering modes, the authors propose to add three modes, thus fully covering all possible spacecraft maneuvers (see Table 1). The new modes are defined for an isolated spacecraft-manipulator system operating in pure weightlessness and in the absence of friction.

**Table 1:** Classification of modes of maneuvering for an isolated spacecraft-manipulator system.

**Case number**	**Commonly used term**	**Proposed terms**	**Linear momentum *P***	**Angular momentum *L***
1	Free-floating	Floating	Conserved^a^	Conserved^a^
2	Free-floating	Rotation-floating^b^	Conserved^a^	Conserved^a^
3	Free-flying	Rotation-flying (translation-floating)	Conserved^a^	Controlled^c^
4	Free-flying	Translation-flying (rotation-floating)	Controlled	Conserved^a^
5	Free-flying	Flying	Controlled	Controlled

a *Apart from the effect of external contact forces (e.g., with the target object)*.

b *The rotation of the base-spacecraft is controlled only by momentum-exchange devices (reaction wheels or control-moment gyroscopes) (Hughes, 2004)*.

c *The rotation of the base-spacecraft is controlled only by external torques (typically provided by reaction-jet thrusters)*.

### Conserved linear and angular momentum

The maneuvering cases here defined as *floating* and *rotation-floating* have in previous literature been referred to as *free-floating*. In both cases the total linear momentum and the total angular momentum of the spacecraft-manipulator system are not subject to any external control forces or torques and are thus conserved.

#### Floating

A spacecraft-manipulator system is here defined to be *floating* when maneuvering in a six DOF under-actuated mode in which only the manipulator joints are actively controlled. The system moves only under the effect of the internal reactions due to the actuation of the manipulator's joint motors. As robotic manipulators mounted on spacecraft typically only use revolute joints, these internal reactions are typically torques. Therefore, the 3 DOF of orientation of the base-spacecraft and the 3 DOF of translation of the system's center-of-mass are not actively controlled.

#### Rotation-floating

A spacecraft-manipulator system is here defined to be *rotation-floating* when maneuvering in a 3 DOF under-actuated mode, in which both the DOF at the manipulator's joints and the three DOF of orientation of the base-spacecraft are controlled by internal torques only. The rotation-floating maneuver case thus differs from floating in that the attitude of the base-spacecraft is actively controlled by momentum-exchange devices [i.e., reaction wheels or control-moment gyroscopes (Hughes, 2004)]. The three DOF of translation of the system's center-of-mass are not actively controlled.

### Controlled linear and angular momentum

All of the three maneuvering cases here defined as *rotation-flying, translation-flying*, and *flying* have been defined in previous literature as *free-flying*. In all three cases, the total angular momentum, total linear momentum, or both are actively controlled by external forces and/or torques, e.g., by a reaction control system.

#### Rotation-flying

A spacecraft-manipulator system is here defined to be *rotation-flying* when the DOF at the manipulator joints are actively controlled by joint motor torques and the three DOF of orientation of the base-spacecraft are actively controlled by external torques only. This is typically achieved by reaction-jet thrusters firing in couples, thus generating a pure torque with total null force. The three DOF of translation of the system center-of-mass are not actively controlled. Therefore, the system's total linear momentum is in this case constant while the angular momentum is time-varying.

#### Translation-flying

A spacecraft-manipulator system is here defined to be *translation-flying* when both the DOF at the manipulator joints and the three DOF of translation of the base-spacecraft are actively controlled. The manipulator DOF are controlled by joint motor torques. The base-spacecraft translation is controlled by external forces, provided typically by reaction-jet thrusters. Furthermore, the three DOF of orientation of the base-spacecraft are either not actively controlled or controlled only by momentum-exchange devices. Therefore, the system's total angular momentum is in this case constant while the linear momentum is time-varying.

#### Flying

A spacecraft-manipulator system is here defined to be *flying* when maneuvering in a mode in which all of the DOF at the manipulator joints are actively controlled by joint motor torques and the six DOF of motion of the base-spacecraft are actively controlled by external forces, provided typically by reaction jet thrusters. In this case, both the system's total angular momentum and the linear momentum are time-varying.

The classification above is rigorously valid only for an isolated spacecraft-manipulator system. In reality, a spacecraft-manipulator system is never isolated but orbiting an extended body (e.g., the Earth) under its gravitational attraction. However, the classification above can still be used in an approximate sense, due to the weightless (e.g., free-falling) condition of the system center-of-mass (due to the balancing of gravitational attraction and centrifugal force) and neglecting the effect of environmental torques (typically dominated by gravity-gradient torque, atmospheric torque, and solar radiation-pressure torque^)^ and non-gravitational environmental forces (typically dominated by atmospheric drag and solar radiation-pressure). The analysis and simulation of *floating, rotation-floating* and *rotation-flying* maneuvering modes can be typically conducted with good accuracy as if the system was isolated. In those three cases, a coordinate system centered at the center-of-mass of the orbiting spacecraft-manipulator system and having axes oriented in a fixed way with respect to an inertial frame (i.e., having zero absolute angular velocity) can be considered as equivalent to an inertial coordinate system for the description of the spacecraft-manipulator system motion.

## Kinematics of a spacecraft-manipulator system

**Figure 1:**
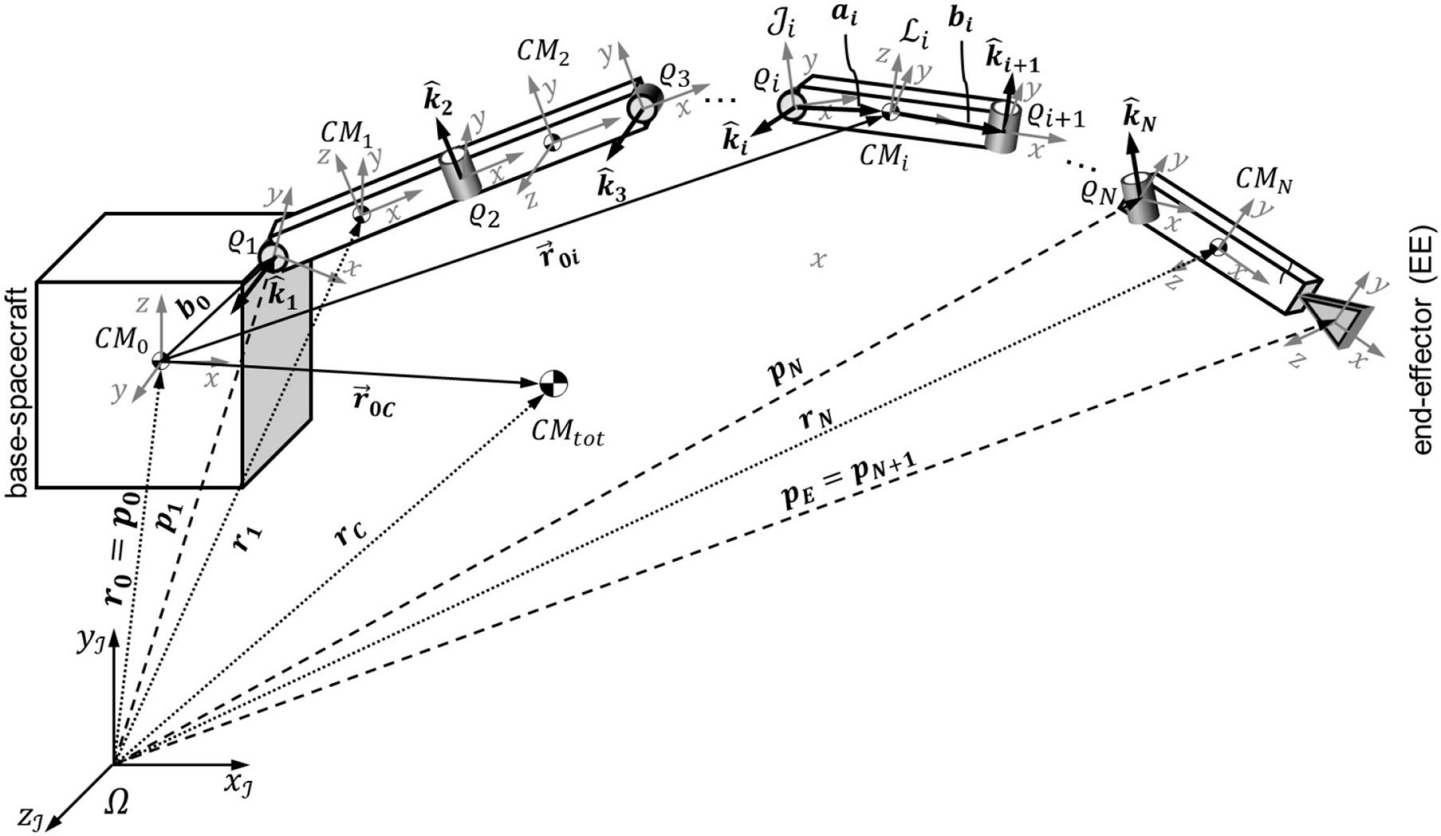
Geometry of a spacecraft-manipulator system.

The geometry of a base-spacecraft equipped with a single *N*-link manipulator is illustrated in Figure 1. The base-spacecraft is defined as link 0, with joint 0 residing at the base-spacecraft center-of-mass. The end-effector (EE) of the manipulator is part of link *N* and located at the end of that link. Therefore, its location can be treated as a virtual joint *N* + *1*. The geometry of a spacecraft-manipulator system with respect to the origin Ω of an inertial reference frame J can be described unequivocally by the position vectors of the joints, ***p***_***i***_ (0 ≤ *i* ≤ *N* + 1), and by the position vectors of the centers-of-mass of the links, ***r***_***j***_ (0 ≤ *j* ≤ *N*). In this paper, the word “vector” is rigorously reserved for Gibbsian vectors (see, for instance, Hughes, 2004).

Three types of Cartesian coordinate systems are used in the analysis: an inertial coordinate system J, a set of *N*+*2* joint-fixed coordinate systems $J_{i}$ (0 ≤ *i* ≤ *N* + 1), and a set of *N* + *1* link-fixed coordinate systems $L_{i}$ (0 ≤ *i* ≤ *N*), with $J_{0}\equiv L_{0}$ and $J_{N+1}=J_{EE}$. The base-spacecraft coordinate system $J_{0}\equiv L_{0}$ has its origin at the center-of-mass of the base-spacecraft, and has an arbitrary orientation (for instance, corresponding to its principal directions of inertia). Each joint coordinate system $J_{i}$ (1 ≤ *i* ≤ *N* + 1) is built using of the Denavit-Hartenberg (DH) convention as specified in Siciliano et al. (2010). The axis $z_{J_{i}}$ of $J_{i}$ is parallel to the joint rotation axis ${\hat{k}}_{i}$ for 1 ≤ *i* ≤ *N*. The origin ϱ_1_ of $J_{1}$ as well as the directions of $x_{J_{1}}$ and $y_{J_{1}}$ can be selected as needed for a particular geometry. The same is true for the end-effector coordinate system $J_{N+1}$. Furthermore, for 2 ≤ *i* ≤ *N*, the origin ϱ_*i*_ of $J_{i}$ is at the intersection of $z_{J_{i}}$ and the common normal between $z_{J_{i-1}}$ and $z_{J_{i}}$. Whenever $z_{J_{i-1}}$ and $z_{J_{i}}$ are parallel, the common normal is uniquely defined and ϱ_*i*_ is selected for convenience (e.g., passing through the center-of-mass of the link *i-1*). The axis $x_{J_{i}}$ points from ϱ_*i*_ along the direction of the common normal defined above. The axis $y_{J_{i}}$ completes the right-handed Cartesian coordinate system. The link coordinate system $L_{i}$ is parallel to $J_{i+1}$ (for 1 ≤ *i* ≤ *N*), but with the origin at the center-of-mass (*CM*_*i*_) of link *i*. This way of defining the joint coordinate systems follows Siciliano with the following difference: Siciliano's link *i* coordinate system is identical to our joint *i* + *1* coordinate system $J_{i+1}$. Furthermore, the origins of the link coordinate systems $L_{i}$ are placed into the link centers-of-mass to comply with Yoshida's approach (Yoshida and Umetani, 1993).

**Figure 2:**
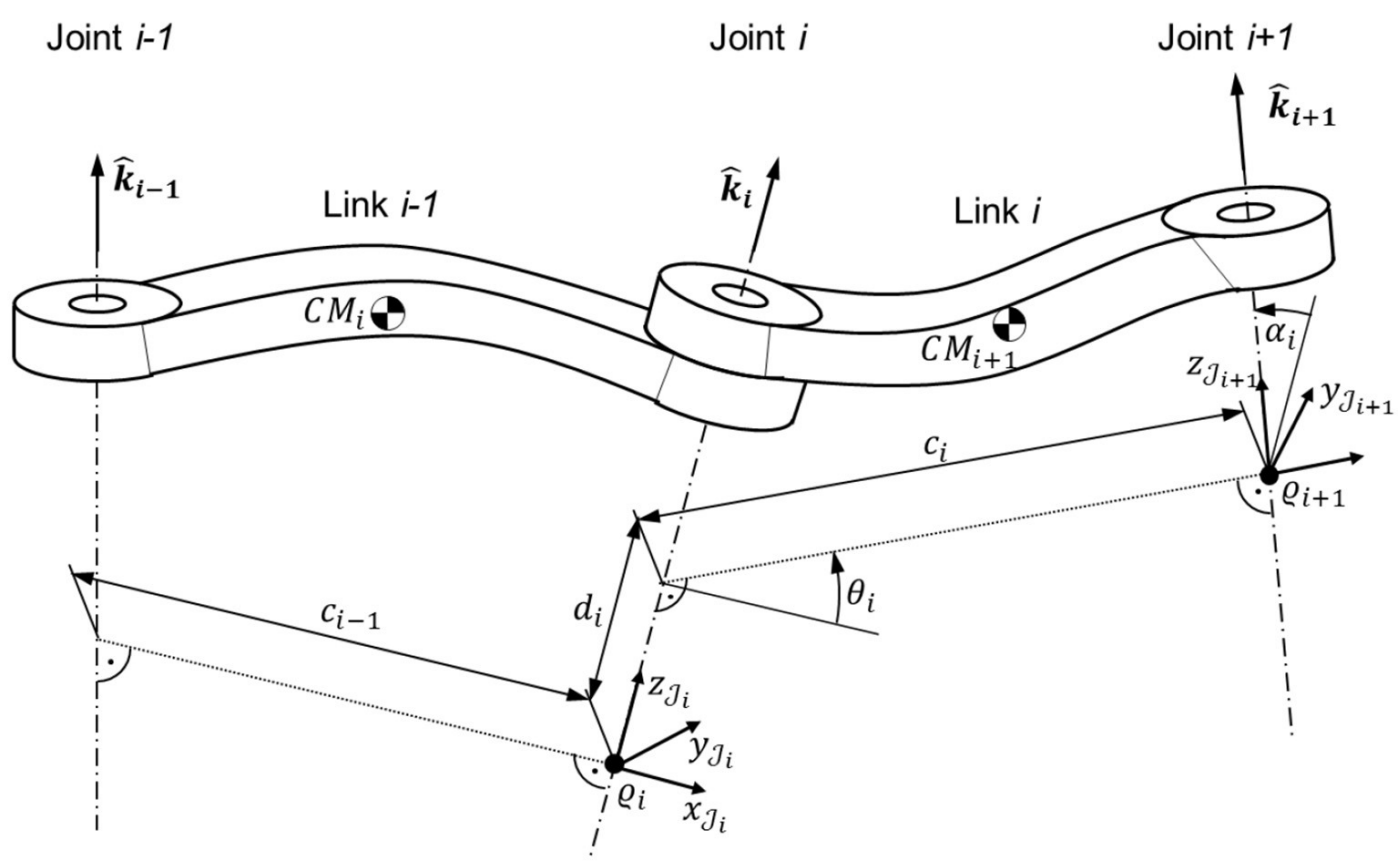
Customized Denavit-Hartenberg kinematic parameters.

The geometrical relationship between subsequent joint coordinate systems $J_{i}$ and $J_{i+1}$ (1 ≤ *i* ≤ *N*) is described by the four DH parameters *d*_*i*_, θ_*i*_, α_*i*_, and *c*_*i*_ (see Table 2 and Figure 2). For a link with a rotary joint, θ_*i*_ is the only variable parameter and is identical to the joint angle *q*_*i*_. The parameters *d*_*i*_, α_*i*_, and *c*_*i*_ represent fixed geometric properties of the manipulator link. For a prismatic joint, the parameter *d*_*i*_ is variable and identical to the joint extension. Since prismatic joints are not commonly used in orbital robotics applications, only rotary joints are considered in this paper.

**Table 2:** Denavit-Hartenberg parameters and their geometric meaning.

**DH parameter**	**Geometric meaning**
*d*_*i*_	Distance between ϱ_*i*_ and $x_{J_{i+1}}$along $z_{J_{i}}$
θ_*i*_	Rotation from $x_{J_{i}}$ to $x_{J_{i+1}}$ about $z_{J_{i}}$
α_*i*_	Rotation from $z_{J_{i}}$ to $z_{J_{i+1}}$ about $x_{J_{i\;+\;1}}$
*c*_*i*_	Distance along the common normal between $z_{J_{i}}$ and $z_{J_{i+1}}$

The homogenous transformation matrix ${\;}^{J_{i}}T_{J_{i+1}}$ from *mathcalJ*_*i*_ to $J_{i+1}$ coordinates can be expressed as a function of the DH parameters as follows:

(1) $${\;}^{J_{i}}T_{J_{i+1}}=A\left(\theta_{i},d_{i},\alpha_{i},c_{i}\right),$$

where the DH transformation matrix function ***A*** (β, *u*, γ, *w*) is defined as:

(2) $$A\left(\beta ,u,\gamma ,w\right)≜\left[\begin{array}{cccc} \text{ cos }\beta & -\text{ sin }\beta \text{ cos }\gamma & \text{ sin }\beta \text{ sin }\gamma & w\text{ cos }\beta \\ \text{ sin }\beta & \text{ cos }\beta \text{ cos }\gamma & -\text{ cos }\beta \text{ sin }\gamma & w\text{ sin }\beta \\ 0 & \text{ sin }\gamma & \text{ cos }\gamma & u\\ 0 & 0 & 0 & 1 \end{array}\right].$$

Therefore, the position and orientation of each joint coordinate system $J_{i}$ (2 ≤ *i* ≤ *N* + 1) can be expressed recursively by a product of DH transformation matrices. The resulting homogenous transformation matrix ${\;}^{J}T_{J_{i}}$ contains the direction-cosine matrix (DCM) ${\;}^{J}R_{J_{i}}$ and the position vector ${\;}^{J}p_{i}$:

(3) $${\;}^{J}T_{J}{\;}_{i}=\left[\begin{array}{cc} {\;}^{J}R_{J_{i}} & {\;}^{J}p_{i}\\ 0_{1,3} & 1 \end{array}\right],$$

Analogously, the homogenous transformation matrix ${\;}^{J}T_{L_{i}}$ contains the DCM ${\;}^{J}R_{L_{i}}$ and position ${\;}^{J}r_{i}$. Notably, because of the fact that $L_{i}$ is oriented as $J_{i+1}$, it yields:

(4) $${\;}^{J}R_{L_{i}}{=}^{J}R_{J_{i+1}}.$$

The joint DCM are used to derive the directions ${\hat{k}}_{i}$ of the joint rotation axes in the inertial frame. In the DH convention, the rotation axis of a rotary joint is defined as the *z* axis in the corresponding link frame. So, ${\hat{k}}_{i}$ is defined by:

(5) $${\;}^{J}{\hat{k}}_{i}{=}^{J}R_{J_{i}}\left(\begin{array}{c} 0\\ 0\\ 1 \end{array}\right).$$

Furthermore, it is useful to define the following additional vectors. The position vector ***a***_***i***_ connects the origin ϱ_*i*_ of the joint *i* coordinate system $J_{i}$ to the center-of-mass of link *i* (CM_i_, origin of link *i* coordinate system $L_{i}$). The position vector ***b***_***i***_ connects CM_i_ and ϱ_*i*+1_. Notably, if CM_i_ lies on a straight line connecting ϱ_*i*_ and ϱ_*i*+1_, which is a valid assumption for most common spacecraft manipulators, ***a***_***i***_ and ***b***_***i***_ can be substituted by their scalar components *a*_*i*_ and *b*_*i*_ on the *x* axis of $L_{i}$ and $J_{i+1}$.

Notably, since the base-spacecraft is designated as link 0 and its center-of-mass is joint 0, it is ***r*_0_ = *p*_0_**, $L_{0}=J_{0}$, ${\;}^{J}R_{L_{0}}{=}^{J}R_{J_{0}}$, and ${\;}^{J}T_{L_{0}}{=}^{J}T_{J_{0}}$. In particular, we have chosen to express the DCM for the base-spacecraft in Euler angles (roll angle ϕ, pitch angle θ, yaw angle ψ) in the following 1-2-3 rotation sequence:

(6) $${\;}^{J}R_{L_{0}}{=}^{J}R_{J_{0}}=\left[\begin{array}{ccc} \text{ cos }\psi & -\text{ sin }\psi & 0\\ \text{ sin }\psi & \text{ cos }\psi & 0\\ 0 & 0 & 1 \end{array}\right]\left[\begin{array}{ccc} \text{ cos }\theta & 0 & \text{ sin }\theta \\ 0 & 1 & 0\\ -\text{ sin }\theta & 0 & \text{ cos }\theta \end{array}\right]\left[\begin{array}{ccc} 1 & 0 & 0\\ 0 & \text{ cos }\phi & -\text{ sin }\phi \\ 0 & \text{ sin }\phi & \text{ cos }\phi \end{array}\right].$$

Therefore:

(7) $${\;}^{J}T_{J}{\;}_{0}{=}^{J}T_{L}{\;}_{0}=\left[\begin{array}{cccc} \text{ cos }\psi \text{ cos }\theta & \text{ cos }\psi \text{ sin }\theta \text{ sin }\psi -\text{ sin }\psi \text{ cos }\psi & \text{ cos }\psi \text{ sin }\theta \text{ cos }\psi +\text{ sin }\psi \text{ sin }\psi & \;\\ \text{ sin }\psi \text{ cos }\theta & \text{ sin }\psi \text{ sin }\theta \text{ sin }\psi +\text{ cos }\psi \text{ cos }\psi & \text{ sin }\psi \text{ sin }\theta \text{ cos }\psi -\text{ cos }\psi \text{ sin }\psi & {\;}^{I}r_{0}\\ -\text{ sin }\theta & \text{ cos }\theta \text{ sin }\psi & \text{ cos }\theta \text{ cos }\psi & \;\\ 0 & 0 & 0 & 1 \end{array}\right].$$

The joint coordinate system $J_{1}$ has a constant position ${\;}^{\text{0}}b_{\text{0}}$ and orientation ${\;}^{L_{0}}R_{J_{1}}$, with respect to the base-spacecraft coordinate system. By expressing ${\;}^{L_{0}}R_{J_{1}}$ in Euler angles with 1-2-3 sequence (ϵ, ζ, and η), it yields:

(8)  JTJ 1=JTJ0J 0TJ1==JTJ 0[cosηcosζcosηsinζsinϵ−sinηcosϵcosηsinζcosϵ+sinηsinϵ sinηcosζ sinηsinζsinϵ+cosηcosϵsinηsinζcosϵ−cosηsinϵ 0b0−sinζ cosζsinϵ cosζcosϵ 0001].

For all of the subsequent joint coordinate systems $J_{i}$, by taking into account Equation (1), it yields:

(9) $$\begin{array}{l} {\;}^{J}T_{J}{\;}_{i}{=}^{J}T_{J}{{\;}_{i-1}}^{J_{i-1}}T_{J_{i}}{=}^{J}T_{J}{\;}_{i-1}A\left(q_{i-1},d_{i-1},\alpha_{i-1},\;a_{i-1}+b_{i-1}\right),\\ \;\forall \;\left\{2\leq i\leq N\right\}, \end{array}$$

where the transformation matrices for the link coordinate systems, ${\;}^{I}T_{L_{i}}$, are derived as:

(10) $${\;}^{J}T_{L}{\;}_{i}{=}^{J}T_{J}{{\;}_{{\;}_{i}}}^{{{\;}_{J}}_{{\;}_{i}}}T_{L_{i}}{=}^{J}T_{J}{\;}_{i}A\left(q_{i},d_{i},\alpha_{i},a_{i}\right),\;\forall \;\left\{1\leq i\leq N\right\}.$$

The relative position between the base-spacecraft center-of-mass and the link centers-of-mass, ${\;}^{J}r_{0i}$, and the position of the system center-of-mass, ${\;}^{J}r_{C}$, can be expressed as:

(11) $${\;}^{J}r_{0i}{=}^{J}r_{i}{-}^{J}\text{​}r_{0},\;\left\{1\leq i\leq N\right\},$$

(12) $$\begin{array}{l} {\;}^{J}r_{C}=\frac{1}{m_{tot}}\sum_{i\;=\;0}^{N}m_{i}{\;}^{J}r_{i}=\frac{1}{m_{tot}}\sum_{i\;=\;0}^{N}m_{i}\;\left({\;}^{J}r_{0}+{\;}^{J}r_{0i}\right)\\ \;={\;}^{J}r_{0}+\frac{1}{m_{tot}}\sum_{i\;=\;1}^{N}m_{i}{\;}^{J}\;r_{0i}, \end{array}$$

where $m_{tot}=\overset{N}{\underset{i=0}{\sum }}m_{i}$.

Furthermore, the position of the system center-of-mass with respect to the base-spacecraft center-of-mass is given by:

(13) $${\;}^{J}r_{0C}={\;}^{J}\text{​}r_{C}-{\;}^{J}\text{​}r_{0}.$$

Equations (12) and (13) yield:

(14) $$m_{tot}{\;}^{J}r_{0C}=\sum_{i=1}^{N}m_{i}{\;}^{J}r_{0i}.$$

Given the geometry of the spacecraft-manipulator system, the linear velocity ${\;}^{J}v_{X_{i}}$ of any point *X*_*i*_ on the *i*-th link of the manipulator and the angular velocity ${\;}^{J}\omega_{i}$ of the *i*-th link can be expressed as:

(15) $${\;}^{J}v_{x_{i}}{=}^{J}v_{0}{+}^{J}\omega_{0}^{\times }\left({\;}^{J}x_{i}{-}^{J}r_{0}\right)+\sum_{k=1}^{i}\left\{\left[{\;}^{J}{\hat{k}}_{k}^{\times }\left({\;}^{J}x_{i}{-}^{J}p_{k}\right)\right]{\dot{q}}_{k}\right\},$$

(16) $${\;}^{J}\omega_{L}{\;}_{i}{=}^{J}\omega_{0}+\sum_{k=1}^{i}\left({\;}^{J}{\hat{k}}_{k}{\dot{q}}_{k}\right).$$

The notation ***a*^×^** is used to denote the skew-symmetric matrix (Hughes, 2004) associated with any vector ***a***, so that the vector product ***c* = *a* × *b*** can be expressed in the matrix-vector operation ***c* = *a*^×^*b***:

(17) $$a^{\times }=\left[\begin{array}{ccc} 0 & -a_{z} & a_{y}\\ a_{z} & 0 & -a_{x}\\ -a_{y} & a_{x} & 0 \end{array}\right].$$

### Alternative expression of transformations using the unit quaternion instead of the DCM

The use of DCM in developing the transformation matrices for the spacecraft-manipulator system is familiar and intuitive. DCM, or equivalently rotation matrices, offer a unique and singularity-free parameterization of the orientation. As such, the DCM method is used above to describe the DH formalism, and DCM-based notation is used throughout this paper. However, the parameterization of the rotation using three parameters, e.g., Euler angle combinations, creates singularities, thus making the resulting DCM non-invertible. In addition, the multiplication of transformation matrices results in a large number of operations, 27 for two [3 × 3] rotation matrices and 64 for two [4 × 4] homogenous transformation matrices, thus making the approach computationally inefficient. The alternative is to use Euler symmetric parameters, also called unit quaternions (Wertz, 1978; Hughes, 2004). Unit quaternions are used to describe rotations following the Euler axis-angle theorem, with the vector part **ε** of the quaternion describing the rotation axis, and the scalar part η describing the rotation angle. A unit quaternion $\bar{q}$ expressing a rotation about any axis $\hat{u}$ by any angle α can be written as a four-vector:

(18) $$\bar{q}=\left[\begin{array}{c} \varepsilon \\ \eta \end{array}\right]=\left[\begin{array}{c} \varepsilon_{1}\\ \varepsilon_{2}\\ \varepsilon_{3}\\ \eta \end{array}\right]=\left[\begin{array}{c} u_{1}\text{ sin }\left(\alpha /2\right)\\ u_{2}\text{ sin }\left(\alpha /2\right)\\ u_{3}\text{ sin }\left(\alpha /2\right)\\ \text{ cos }\left(\alpha /2\right) \end{array}\right].$$

The magnitude of $\bar{q}$ is $|\bar{q}|=1$, hence $\bar{q}$ is called a unit quaternion. The unit quaternion thus corresponds to a rotation matrix ***R*** (Hughes, 2004):

(19) $$R=\left[\begin{array}{ccc} 1-2\left(\varepsilon_{2}^{2}+\varepsilon_{3}^{2}\right) & 2(\varepsilon_{1}\varepsilon_{2}-\varepsilon_{3}\eta ) & 2(\varepsilon_{1}\varepsilon_{3}+\varepsilon_{2}\eta )\\ 2(\varepsilon_{1}\varepsilon_{2}+\varepsilon_{3}\eta ) & 1-2(\varepsilon_{1}^{2}+\varepsilon_{3}^{2}) & 2(\varepsilon_{2}\varepsilon_{3}-\varepsilon_{1}\eta )\\ 2(\varepsilon_{1}\varepsilon_{3}-\varepsilon_{2}\eta ) & 2(\varepsilon_{2}\varepsilon_{3}+\varepsilon_{1}\eta ) & 1-2(\varepsilon_{1}^{2}+\varepsilon_{2}^{2}) \end{array}\right].$$

A sequence of rotations, ***R*_3_ = *R*_1_*R*_2_**, is then expressed by the quaternion product (Campa and Camarillo, 2008):

(20) $${\bar{q}}_{3}={\bar{q}}_{1}⊗{\bar{q}}_{2}=\left[\begin{array}{c} \eta_{1}\varepsilon_{2}+\eta_{2}\varepsilon_{1}+\varepsilon_{1}^{\times }\varepsilon_{2}\\ \eta_{1}\eta_{2}-\varepsilon_{1}^{T}\varepsilon_{2} \end{array}\right].$$

Therefore, any vector ***a***, expressed as four-vector $\text{a}={\begin{array}{cccc} {[a}_{1} & a_{2} & a_{3} & 0] \end{array}}^{T}$, can be rotated using a unit quaternion (Kuipers, 2000):

(21) $$a\prime =\bar{q}⊗a⊗{\bar{q}}^{*},$$

with the conjugate quaternion ${\bar{q}}^{*}={\begin{array}{cc} {}[-{\varepsilon ]}^{T} & \eta \end{array}}^{T}$. Therefore, the application of the DH convention results in an unit quaternion ${\;}^{J_{i}}{{\bar{q}}_{r}}_{J_{i+\text{1}}}$ for the rotation between coordinate systems $J_{i}$ and $J_{i+1}$, and a four-vector ${\;}^{J_{i}}t_{J_{i+1}}$ for the translation between origins of $J_{i}$ and $J_{i+1}$ (Campa and Camarillo, 2008):

(22) $${\;}^{J_{i}}{\bar{q}}_{r}{\;}_{J_{i+1}}=\left[\begin{array}{c} \text{ cos }\frac{\theta_{i}}{2}\text{ sin }\frac{\alpha_{i}}{2}\\ \;\\ \text{ sin }\frac{\theta_{i}}{2}\text{ cos }\frac{\alpha_{i}}{2}\\ \text{ cos }\frac{\theta_{i}}{2}\text{ cos }\frac{\alpha_{i}}{2} \end{array}\right],$$

(23) $${\;}^{J_{i}}t_{J_{i+1}}=\left[\begin{array}{c} c_{i}\text{ cos }\theta_{i}\\ c_{i}\text{ sin }\theta_{i}\\ d_{i}\\ 0 \end{array}\right].$$

The rotation from the base joint $J_{1}$ to the end-effector $J_{N+1}$ can thus be computed from a sequence of rotations using Equation (20):

(24) $${\;}^{1}{\bar{q}}_{r}{\;}_{E}{=}^{1}{\bar{q}}_{r}{\;}_{N+1}{=}^{1}{\bar{q}}_{r}{\;}_{2}{⊗}^{2}{\bar{q}}_{r}{\;}_{3}⊗\ldots {⊗}^{N-1}{\bar{q}}_{r}{\;}_{N}⊗{\;}^{N}{\bar{q}}_{r}{\;}_{N+1}.$$

The sequence of translations results from Equation (21):

(25) $$\begin{array}{l} {\;}^{1}t_{E}{=}^{1}t_{N+1}{=}^{1}t_{2}+\\ \;{+}^{1}{\bar{q}}_{r}{\;}_{2}{⊗}^{2}t_{3}{⊗}^{1}{\bar{q}}_{r_{2}}^{*}+\\ \;+{(}^{1}{\bar{q}}_{r}{\;}_{2}{⊗}^{2}{\bar{q}}_{r}{\;}_{3}){⊗}^{3}t_{4}⊗{\left({\;}^{1}{\bar{q}}_{r}{\;}_{2}{⊗}^{2}{\bar{q}}_{r}{\;}_{3}\right)}^{*}+\ldots +\\ \;+{(}^{1}{\bar{q}}_{r}{\;}_{2}⊗\ldots {⊗}^{N-1}{\bar{q}}_{r}{\;}_{N}){⊗}^{N}t_{N+1}\\ \;⊗{\left({\;}^{1}{\bar{q}}_{r}{\;}_{2}⊗\ldots {⊗}^{N-1}{\bar{q}}_{r}{\;}_{N}\right)}^{*}. \end{array}$$

## Dynamics of a floating spacecraft-manipulator system

The equations of motion of the spacecraft-manipulator system illustrated in Figure 1 are here derived using the Lagrangian approach for the case of floating maneuvering mode (see section Detailed Classification of Spacecraft-Manipulator System Maneuvering and Table 1). In this case, the potential energy of the system is zero, and the Lagrangian equals the kinetic energy *T*:

(26) $$L=T=\frac{1}{2}\sum_{i\;=\;0}^{N}\left({\;}^{J}\omega_{i}{{\;}^{T}}^{J}I_{i}{\;}^{J}\omega_{i}+m_{i}{\;}^{J}{\dot{r}}_{i}{{\;}^{T}}^{J}{\dot{r}}_{i}\right).$$

By collecting the mass and inertia properties of the spacecraft-manipulator system into the [6 × 6] base-spacecraft inertia matrix ***H*_0_**, the [*N* × *N*]manipulator inertia matrix ***H*_*m*_**, and the [6 × *N*] dynamic-coupling inertia matrix ***H*_*0m*_**, after some algebraic steps the kinetic energy can be expressed as (Yoshida and Umetani, 1993):

(27) $$\begin{array}{l} T=\frac{1}{2}\left[{\;}^{J}{\dot{x}}_{0}^{T}{\dot{q}}^{T}\right]\left[\begin{array}{cc} H_{0} & H_{0m}\\ H_{0m}^{T} & H_{m} \end{array}\right]\left[\begin{array}{c} {\;}^{J}{\dot{x}}_{0}\\ \dot{q} \end{array}\right]\\ \;={\frac{1}{2}}^{J}{\dot{x}}_{0}^{T}H_{0}{\;}^{J}{\dot{x}}_{0}+\frac{1}{2}{\dot{q}}^{T}H_{m}\dot{q}\\ \;+{\frac{1}{2}}^{J}{\dot{x}}_{0}TH_{0m}\dot{q}+\frac{1}{2}{\dot{q}}^{T}H_{0m}^{T}{\;}^{J}{\dot{x}}_{0}, \end{array}$$

where ${\;}^{J}{\dot{x}}_{0}={\left[\begin{array}{cc} {\;}^{J}v_{0}{\;}^{T} & {\;}^{J}\omega_{0}^{T} \end{array}\right]}^{T}$ is the combined and angular velocity matrix of the base-spacecraft. The base-spacecraft inertia matrix, manipulator inertia matrix, and the dynamic-coupling inertia matrix are thoroughly explained in the following subsections.

### Base-spacecraft inertia matrix

The [6 × 6] base-spacecraft inertia matrix ***H*_0_** is resulting to be:

(28) $$H_{0}=\left[\begin{array}{cc} m_{tot}\;{𝕀}_{3,3} & -m_{tot}{\;}^{J}r_{0C}^{\times }\\ m_{tot}{\;}^{J}r_{0C}^{\times } & H_{S} \end{array}\right],$$

where 𝕀_**3, 3**_ is the [3 × 3] identity matrix and the [3 × 3] submatrix ***H*_*S*_** collects the moments-of-inertia of the spacecraft-manipulator system about the base-spacecraft center-of-mass, expressed in the inertial frame J:

(29) $$H_{S}=\sum_{i=1}^{N}\left({\;}^{J}I_{i}-m_{i}{\;}^{J}r_{0i}^{\times }{\;}^{J}r_{0i}^{\times }\right){+}^{J}I_{0}.$$

The *i*-th link moments-of-inertia matrix in the inertial coordinate system is derived from the moments-of-inertia matrix in the *i*-th link coordinate system as:

(30) $${\;}^{J}I_{i}{=}^{J}R_{L_{i}}{\;}^{L_{i}}I_{i}{\;}^{J}R_{L_{i}}^{T}.$$

### Dynamic-coupling inertia matrix

The [6 × *N*] dynamic-coupling inertia matrix ***H*_*0m*_** expresses the contribution of the dynamic coupling between the manipulator and the base-spacecraft to the kinetic energy of the spacecraft-manipulator system. In particular, it is:

(31) $$H_{0m}=\left[\begin{array}{c} J_{TS}\\ H_{Sq} \end{array}\right],$$

where the [3 × *N*] submatrix J**_*TS*_** collects the contribution to the system kinetic energy of the combination of the effect of the manipulator joint rate $\dot{q}$ and the base-spacecraft linear velocity ${\;}^{J}v_{0}$. In detail, it is:

(32) $$J_{TS}=\sum_{i=1}^{N}\left(m_{i}J_{Ti}\right),$$

where the [3 × *N*] matrix ***J*_*Ti*_** represents the linear velocity Jacobian for the center-of-mass of the *i*-th link (Siciliano et al., 2010), and is given by:

(33) $$\begin{array}{l} J_{Ti}=\left[\begin{array}{cccc} {\;}^{I}{\hat{k}}_{1}^{\times }\left({\;}^{J}r_{i}{-}^{J}p_{1}\right) & \cdots & {\;}^{I}{\hat{k}}_{i}^{\times }\left({\;}^{J}r_{i}{-}^{J}p_{i}\right) & 0_{3,N-i} \end{array}\right],\\ \;\forall \;\left(1\leq i\leq N\right). \end{array}$$

Finally, the [3 × *N*] submatrix ***H*_*Sq*_** contains the contribution to the system kinetic energy of the combination of the effect of the manipulator joint rate $\dot{q}$ and the base-spacecraft angular velocity ${\;}^{J}\omega_{0}$. In detail, it is:

(34) $$H_{Sq}=\overset{N}{\underset{i=1}{\sum }}\left({\;}^{J}I_{i}J_{Ri}+m_{i}{\;}^{J}r_{0i}^{\times }J_{Ti}\right),$$

where the [3 × *N*] matrix J_***Ri***_ represents the angular velocity Jacobian for the *i*-th link (Siciliano et al., 2010):

(35) $$J_{Ri}=\left[\begin{array}{cccc} {\;}^{J}{\hat{k}}_{1} & \cdots & {\;}^{J}{\hat{k}}_{i} & 0_{3,N-i} \end{array}\right],\;\forall \;\left(1\leq i\leq N\right).$$

### Manipulator inertia matrix

The [*N* × *N*] inertia matrix for the manipulator, ***H*_*m*_**, is identical to that of any fixed-base manipulator (Siciliano et al., 2010) and expressed as:

(36) $$H_{m}=\sum_{i=1}^{N}\left(J_{Ri}^{T}{\;}^{J}I_{i}J_{Ri}+m_{i}J_{Ti}^{T}J_{Ti}\right).$$

### Equations of motion of a floating spacecraft-manipulator system

The generalized coordinates for the description of the spacecraft-manipulator system were chosen to be the manipulator joint angles ***q*** and the base-spacecraft combined linear and angular position ${\;}^{J}x_{0}$, resulting in the following two matricial Lagrangian equations. In the following, the coordinate system superscript will be omitted for better readability:

(37) $$\frac{d}{dt}\left(\frac{\partial T}{\partial {\dot{x}}_{0}}\right)-\frac{\partial T}{\partial x_{0}}=0_{6,1},$$

(38) $$\frac{d}{dt}\left(\frac{\partial T}{\partial \dot{q}}\right)-\frac{\partial T}{\partial q}=\tau ,$$

where the [*N* × 1] matrix **τ** contains the manipulator joint torques. It would be possible to include the effects of the internal torques due to the presence of momentum-exchange devices on the right-hand side of Equation (37), following the procedure outlined in Wie (2008). That would extend the analysis to the case of *rotation-floating* (see Table 1).

By substituting the kinetic energy expressed in Equation (27) into Equations (37) and (38), by computing the derivatives, and by properly rearranging the terms, the matricial equations of motion (corresponding to *N*+*6* scalar equations) for the floating spacecraft-manipulator system result as:

(39) $$\left[\begin{array}{cc} H_{0} & H_{0m}\\ H_{0m}^{T} & H_{m} \end{array}\right]\left[\begin{array}{c} {\overset{..}{x}}_{0}\\ \overset{..}{q} \end{array}\right]+\left[\begin{array}{cc} {\dot{H}}_{0} & {\dot{H}}_{0m}\\ {\dot{H}}_{0m}^{T} & {\dot{H}}_{m} \end{array}\right]\left[\begin{array}{c} {\dot{x}}_{0}\\ \dot{q} \end{array}\right]+\left[\begin{array}{c} c_{0}\\ c_{m} \end{array}\right]=\left[\begin{array}{c} 0_{6,1}\\ \tau \end{array}\right],$$

where the [6 × 1] matrix ***c*_0_** and the [*N* × 1] matrix ***c*_*m*_** are defined as:

(40) $$c_{0}=-\frac{\partial T}{\partial x_{0}}=-\frac{1}{2}\frac{\partial }{\partial x_{0}}\left({\dot{x}}_{0}^{T}H_{0}{\dot{x}}_{0}+{\dot{q}}^{T}H_{m}\dot{q}+{\dot{x}}_{0}^{T}H_{0m}\dot{q}+{\dot{q}}^{T}H_{0m}^{T}{\dot{x}}_{0}\right),$$

(41) $$c_{m}=-\frac{\partial T}{\partial q}=-\frac{1}{2}\frac{\partial }{\partial q}\left({\dot{x}}_{0}^{T}H_{0}{\dot{x}}_{0}+{\dot{q}}^{T}H_{m}\dot{q}+{\dot{x}}_{0}^{T}H_{0m}\dot{q}+{\dot{q}}^{T}H_{0m}^{T}{\dot{x}}_{0}\right).$$

## Generalized form of the equations of motion for a floating spacecraft-manipulator system

The equations of motion in Equation (39) govern the dynamics of a floating spacecraft-manipulator system in terms of coupled base-spacecraft motion (first six scalar equations) and manipulator motion (last *N* scalar equations). In this section, the *N*+*6* scalar equations of motion are rewritten into a set of *N* scalar generalized equations, as typically done for under-actuated systems (Yoshida and Nenchev, 1998). The linear momentum, ***P***, and the angular momentum, ***L***, are given by (Umetani and Yoshida, 1989):

(42) $$\left[\begin{array}{c} P\\ L \end{array}\right]=H_{0}{\dot{x}}_{0}+H_{0m}\dot{q}=M_{0},$$

where $M_{0}={\left[\begin{array}{cc} P & L \end{array}\right]}^{T}$ is the [61] combined matrix of initial linear and angular momentum about the base-spacecraft center-of-mass, expressed in the inertial frame J. Because of the hypothesis of floating maneuvering in the absence of any external forces or moments, the location of the system center-of-mass remains constant, the momenta are conserved, and therefore it yields:

(43) $$\frac{d}{dt}\left(\begin{array}{c} P\\ L \end{array}\right)=H_{0}{\overset{..}{x}}_{0}+H_{0m}\overset{..}{q}+{\dot{H}}_{0}{\dot{x}}_{0}+{\dot{H}}_{0m}\dot{q}=0.$$

Equation (42) is solved for ${\dot{x}}_{0}$. The result is inserted into Equation (43), which is then solved for ${\ddot{x}}_{0}$. The resulting expressions are finally inserted into the last *N* scalar equations of Equation (39). By considering the symmetry of $H_{0}^{-\text{1}}$ (which will be discussed below), the resulting matricial equation (corresponding to *N* scalar equations) is:

(44) $$\begin{array}{l} H^{⋆}\overset{..}{q}+H^{\cdot ⋆}\dot{q}-\frac{1}{2}\frac{\partial }{\partial q}\left({\dot{q}}^{T}H^{⋆\dot{q}}\right)=\tau -\frac{d}{dt}\left(H_{0m}^{T}H_{0}^{-1}\right)M_{0}\\ \;+\frac{1}{2}\frac{\partial }{\partial q}\left(M_{0}^{T}H_{0}^{-T}M_{0}\right), \end{array}$$

where the [*N* × *N*] generalized inertia matrix ***H*^⋆^** is defined to be:

(45) $$H^{⋆}=H_{m}-H_{0m}^{T}H_{0}^{-1}H_{0m}.$$

The further treatment of this generalized equation of motion in the presence on non-zero angular momentum is described in Nanos and Papadopoulos (2011). For the purposes of this tutorial, we assume from now on that the initial momentum of the floating spacecraft-manipulator system is zero, i.e.:

(46) $$M_{0}=0_{6,1}.$$

Therefore, Equation (44) simplifies to:

(47) $$H^{⋆}\overset{..}{q}+H^{\cdot ⋆}\dot{q}+c^{⋆}=\tau ,$$

where the [*N* × 1] matrix *c*^⋆^ is defined as:

(48) $$c^{⋆}=-\frac{1}{2}\frac{\partial }{\partial q}\left({\dot{q}}^{T}H^{⋆}\dot{q}\right)=-\frac{1}{2}{\dot{q}}^{T}\frac{\partial H^{⋆}}{\partial q}\dot{q}.$$

The velocity and position dependent terms in the generalized equations of motion can be combined into a single [*N* × 1] matrix ***C*^⋆^**:

(49) $$C^{⋆}\left(q,\dot{q}\right)=H{\dot{\;}}^{⋆}\dot{q}+c^{⋆}.$$

This permits the familiar representation of the generalized equations of motion which closely resembles the equations of motion for a fixed-base manipulator (Siciliano et al., 2010):

(50) $$H^{⋆}\overset{..}{q}+C^{⋆}(q,\dot{q})=\tau .$$

The thorough symbolic expression of the terms of Equation (47) is provided in the following subsections.

### Inverse base-spacecraft inertia matrix

The calculation of the symbolic expression of ***H***^⋆^ from Equation (45) requires the inverse of the [6 × 6] base-spacecraft inertia matrix ***H***_**0**_. The matrix ***H***_**0**_ is a partitioned block matrix of the form:

(51) $$H_{0}=\left[\begin{array}{cc} m_{tot}{𝕀}_{3,3} & -m_{tot}r_{0C}^{\times }\\ m_{tot}r_{0C}^{\times } & H_{S} \end{array}\right]=\left[\begin{array}{cc} U & V\\ W & X \end{array}\right].$$

By following the approach taken by Mukherjee and Nakamura (1992), the inverse of ***H***_**0**_ is determined using the Banachiewicz inversion formula (Baksalary and Styan, 2002) and the Schur complement ***S***_***U***_ of the non-singular submatrix ***U***:

(52) $$\begin{array}{l} H_{0}^{-1}=\left[\begin{array}{cc} {𝕀}_{3,3} & -U^{-1}V\\ 0_{3,3} & {𝕀}_{3,3} \end{array}\right]\left[\begin{array}{cc} U^{-1} & 0_{3,3}\\ 0_{3,3} & S_{U}^{-1} \end{array}\right]\left[\begin{array}{cc} {𝕀}_{3,3} & 0_{3,3}\\ -WU^{-1} & {𝕀}_{3,3} \end{array}\right]\\ \;=\left[\begin{array}{cc} \frac{1}{m_{tot}}{𝕀}_{3,3}-r_{0C}^{\times }S_{U}^{-1}r_{0C}^{\times } & r_{0C}^{\times }S_{U}^{-1}\\ S_{U}^{-1}r_{0C}^{\times } & S_{U}^{-1} \end{array}\right], \end{array}$$

where the Schur complement of ***U*** for a matrix $\left[\begin{array}{cc} U & V\\ W & X \end{array}\right]\;$ is defined as Baksalary and Styan (2002):

(53) $$S_{U}=X-WU^{-1}V=H_{S}+m_{tot}r_{0C}^{\times }r_{0C}^{\times }.$$

In detail, ***S***_***U***_ is a symmetric [3 × 3] matrix with the elements:

(54) $$s_{1,1}=\;\sum_{i=1}^{N}\left(I_{xx_{i}}+m_{i}\left(r_{0i_{y}}^{2}+r_{0i_{z}}^{2}\right)\right)+I_{xx_{0}}-m_{tot}\left(r_{0C_{y}}^{2}+r_{0C_{z}}^{2}\right),$$

(55) $$s_{1,2}=s_{2,1}=\sum_{i=1}^{N}\left(I_{xy_{i}}-m_{i}r_{0i_{x}}r_{0i_{y}}\right)+I_{xy_{0}}+m_{tot}r_{0C_{x}}r_{0C_{y}},$$

(56) $$s_{1,3}=s_{3,1}=\sum_{i=1}^{N}\left(I_{xz_{i}}-m_{i}r_{0i_{x}}r_{0i_{z}}\right)+I_{xz_{0}}+m_{tot}r_{0C_{x}}r_{0C_{z}},$$

(57) $$s_{2,2}=\;\sum_{i=1}^{N}\left(I_{yy_{i}}+m_{i}\left(r_{0i_{y}}^{2}+r_{0i_{z}}^{2}\right)\right)+I_{yy_{0}}-m_{tot}\left(r_{0C_{x}}^{2}+r_{0C_{z}}^{2}\right),$$

(58) $$s_{2,3}=s_{3,2}=\sum_{i=1}^{N}\left(I_{yz_{i}}-m_{i}r_{0i_{y}}r_{0i_{z}}\right)+I_{yz_{0}}+m_{tot}r_{0C_{y}}r_{0C_{z}},$$

(59) $$s_{3,3}=\;\sum_{i=1}^{N}\left(I_{zz_{i}}+m_{i}\left(r_{0i_{y}}^{2}+r_{0i_{z}}^{2}\right)\right)+I_{zz_{0}}-m_{tot}\left(r_{0C_{x}}^{2}+r_{0C_{y}}^{2}\right).$$

The determinant of ***S***_***U***_ is given by:

(60) $$\begin{array}{l} \det\left(S_{U}\right)=-s_{13}^{2}s_{22}+2s_{12}s_{13}s_{23}-s_{11}s_{23}^{2}-s_{12}^{2}s_{33}\\ \;+\;s_{\text{11}}s_{\text{22}}s_{\text{33}}\text{.} \end{array}$$

Therefore, ***S***_***U***_ can be inverted symbolically, resulting in the symmetric [3 × 3] matrix:

(61) $$\begin{array}{l} S_{U}^{-1}=\\ \;\frac{1}{det\left(S_{U}\right)}\left[\begin{array}{ccc} s_{22}s_{33}-s_{23}^{2} & s_{13}s_{23}-s_{12}s_{33} & s_{12}s_{23}-s_{13}s_{22}\\ s_{13}s_{23}-s_{12}\;s_{33} & s_{11}s_{33}-s_{13}^{2} & s_{12}s_{13}-s_{11}s_{23}\\ s_{12}s_{23}-s_{13}s_{22} & s_{12}s_{13}-s_{11}s_{23} & s_{11}s_{22}-s_{12}^{2} \end{array}\right] \end{array}$$

This symbolic expression for ***S***_***U***_ allows the calculation of the symbolic expression of $H_{0}^{-\text{1}}$ by using Equation (52).

### Time derivative of the generalized inertia matrix

The time derivative of the generalized inertia matrix, ${\dot{H}}^{⋆}$, is calculated by taking the time derivative of Equation (45), resulting in:

(62) $${\dot{H}}^{⋆}=\frac{d}{dt}\left(H_{m}-H_{0m}^{T}H_{0}^{-1}H_{0m}\right).$$

The derivative of a matrix inverse with respect to a scalar can be expressed as:

(63) $$\frac{dQ^{-1}}{dx}=-Q^{-1}\frac{dQ}{dx}Q^{-1},$$

as it can be immediately obtained from

(64) $$\frac{d\left(QQ^{-1}\right)}{dx}=\frac{dE}{dx}=0=\frac{dQ}{dx}Q^{-1}+Q\frac{dQ^{-1}}{dx},$$

where **Q** is any square-invertible matrix and *x* is a scalar. This allows computing the time derivative of the generalized inertia matrix without the need for calculating the time derivative of the inverse of the base-spacecraft inertia matrix, $H_{\text{0}}^{-\text{1}}$:

(65) $$\begin{array}{l} {\dot{H}}^{⋆}={\dot{H}}_{m}-({\dot{H}}_{0m}^{T}H_{0}^{-1}H_{0m}+H_{0m}^{T}H_{0}^{-1}{\dot{H}}_{0m}\\ \;\;-\;H_{0m}^{T}H_{0}^{-1}{\dot{H}}_{0}H_{0}^{-1}H_{0m}). \end{array}$$

The following subsections discuss the calculation of the individual matrix derivatives in Equation (65).

#### Time derivative of the manipulator inertia matrix

By taking the time derivative of Equation (36), it yields:

(66) $$\begin{array}{l} {\dot{H}}_{m}=\sum_{i=1}^{N}({\dot{J}}_{Ri}^{T}{\;}^{J}I_{i}J_{Ri}+J_{Ri}^{T}{\;}^{J}{\dot{I}}_{i}J_{Ri}+J_{Ri}^{T}{\;}^{J}I_{i}{\dot{J}}_{Ri}\\ \;+\;m_{i}\left({\dot{J}}_{Ti}^{T}\;J_{Ti}+J_{Ti}^{T}\;{\dot{J}}_{Ti}\right)) \end{array}$$

The only new values to be calculated are the time derivatives of the angular and linear motion Jacobians, ${\dot{J}}_{Ri}$ and ${\dot{J}}_{Ti}$, as well as the time derivative of the link moments-of-inertia matrices, ${\;}^{J}{\dot{I}}_{i}$ (1 ≤ *i* ≤ *N*). For the calculation of these terms, the time derivatives of the joint and link direction-cosine matrices, ${\;}^{J}{\dot{R}}_{J_{i}}$ and ${\;}^{J}{\dot{R}}_{L_{i}}$ are required. The time derivative of any joint direction-cosine matrix ${\;}^{J}R_{J_{\text{i}}}$ is given by the Darboux equation (Hughes, 2004):

(67) $${\;}^{J}{\dot{R}}_{J_{i}}{=}^{J}\omega_{J_{i}}^{\times }{\;}^{J}R_{J_{i}}.$$

The angular velocity of any joint coordinate system with respect to the inertial frame can be computed recursively from:

(68) $$\begin{array}{l} {\;}^{J}\omega_{J_{i}}{=}^{J}\omega_{J_{i-1}}{+}^{i-1}\omega_{J_{i}}{=}^{J}\omega_{J_{i-1}}{+}^{J}\hat{k}{\;}_{J_{i-1}}{\dot{q}}_{i-1},\\ \;\forall \;\left(1\leq i\leq N+1\right). \end{array}$$

Due to the definition of the joint and link coordinate systems in section Kinematics of a Spacecraft-Manipulator System:

(69) $${\;}^{J}{\dot{R}}_{L_{i}}{=}^{J}{\dot{R}}_{J_{i+1}},\;\forall \;\left(1\leq i\leq N\right).$$

Furthermore, the time derivative of Equation (30) yields:

(70) $$\begin{array}{l} {\;}^{J}{\dot{I}}_{i}{=}^{J}{\dot{R}}_{L_{i}}{\;}^{L_{i}}I_{i}{\;}^{J}R_{L_{i}}^{T}{+}^{J}R_{L_{i}}{\;}^{L_{i}}I_{i}{\;}^{J}{\dot{R}}_{L_{i}}^{T},\\ \;\forall \;\left(1\leq i\leq N\right). \end{array}$$

The time derivative of the angular velocity Jacobian defined in Equation (35) is:

(71) $$\begin{array}{l} {\dot{J}}_{Ri}=\left[\begin{array}{ccccc} {\;}^{J}{\dot{R}}_{J_{1}}\left(\begin{array}{c} 0\\ 0\\ 1 \end{array}\right) & {\;}^{J}{\dot{R}}_{J_{2}}\left(\begin{array}{c} 0\\ 0\\ 1 \end{array}\right) & \ldots & {\;}^{J}{\dot{R}}_{J_{i}}\left(\begin{array}{c} 0\\ 0\\ 1 \end{array}\right) & 0_{3,N-1} \end{array}\right],\\ \;\forall \;\left(1\leq i\leq N\right). \end{array}$$

The time derivative of the linear velocity Jacobian defined in Equation (33) is computed from:

(72) $${\dot{J}}_{Ti}=\left[\begin{array}{ccccc} {\dot{j}}_{Ti_{1}} & {\dot{j}}_{Ti_{2}} & \cdots & {\dot{j}}_{Ti_{i}} & 0_{3,N-1} \end{array}\right],\;\forall \;\left(1\leq i\leq N\right),$$

where:

(73) $${\dot{j}}_{Ti_{k}}=\begin{array}{c} {\left[{\;}^{J}{\dot{R}}_{J_{k}}\left(\begin{array}{c} 0\\ 0\\ 1 \end{array}\right)\right]}^{\times }\left(r_{i}-p_{k}\right)+{\hat{k}}_{k}^{\times }\left({\dot{r}}_{i}-{\dot{p}}_{k}\right),\;\forall \;\left(k\leq i\right) \end{array}.$$

#### Time derivative of the base-spacecraft inertia matrix

The time derivative of Equation (28) is given as:

(74) $${\dot{H}}_{0}=\left[\begin{array}{cc} 0_{3,3} & -m_{tot}{\dot{r}}_{0C}^{\times }\\ m_{tot}{\dot{r}}_{0C}^{\times } & {\dot{H}}_{S} \end{array}\right].$$

By using Equations (11) and (13), the upper right and lower left sub-matrices of Equation (74) can be obtained from the following:

(75) $$\begin{array}{l} m_{tot}{\dot{r}}_{0C}^{\times }=\sum_{i=1}^{N}m_{i}{\dot{r}}_{0i}^{\times }=\sum_{i=1}^{N}m_{i}{\left({\dot{r}}_{i}-{\dot{r}}_{0}\right)}^{\times }=\sum_{i=1}^{N}m_{i}\\ \;{\left(\omega_{0}{\;}^{\times }\left(r_{i}-r_{0}\right)+\sum_{j=1}^{i}\left\{\left[{\left[{\;}^{J}R_{J_{j}}\left(\begin{array}{c} 0\\ 0\\ 1 \end{array}\right)\right]}^{\times }\left(r_{i}-p_{j}\right)\right]{\dot{q}}_{j}\right\}\right)}^{\times }. \end{array}$$

The lower right sub-matrix of Equation (74) is computed by using Equation (29):

(76) $${\dot{H}}_{S}=\sum_{i=1}^{N}\left({\;}^{J}{\dot{I}}_{i}-m_{i}\left({\dot{r}}_{0i}^{\times }r_{0i}^{\times }+r_{0i}^{\times }{\dot{r}}_{0i}^{\times }\right)\right){+}^{J}{\dot{I}}_{0}.$$

#### Time derivative of the dynamic-coupling inertia matrix

The time derivative of the dynamic-coupling matrix *H*_0*m*_ in Equation (31) results in:

(77) $${\dot{H}}_{0m}=\left[\begin{array}{c} {\dot{J}}_{TS}\\ {\dot{H}}_{Sq} \end{array}\right],$$

where from Equations (32) and (34):

(78) $${\dot{J}}_{TS}=\sum_{i=1}^{N}m_{i}{\dot{J}}_{Ti},$$

(79) $${\dot{H}}_{Sq}=\sum_{i=1}^{N}\left({\;}^{J}{\dot{I}}_{i}J_{Ri}{+}^{J}I_{i}{\dot{J}}_{Ri}+m_{i}\left({\dot{r}}_{0i}^{\times }J_{Ti}+r_{0i}^{\times }{\dot{J}}_{Ti}\right)\right).$$

### Expression of the matrix *c*^⋆^

From Equation (48), the elements of ***c***^**⋆**^ can be expressed as:

(80) $$c_{k}^{⋆}=-\frac{1}{2}{\dot{q}}^{T}\frac{\partial H^{⋆}}{\partial q_{k}}\dot{q},\;\forall \;\left(1\leq k\leq N\right),$$

For the planar case, Papadopoulos (1990) provides an expression for ***c***^**⋆**^. For the general case, the symbolic computation of *c*^⋆^ requires the derivatives of ***H***^**⋆**^ with respect to each joint angle ***q***_***k***_. By following the analogous procedure as used for the time derivative of ***H***^**⋆**^ (see Equation 65), it yields:

(81) $$\begin{array}{l} \frac{\partial H^{⋆}}{\partial q_{k}}=\frac{\partial H_{m}}{\partial q_{k}}-{\frac{\partial H_{0m}}{\partial q_{k}}}^{T}H_{0}^{-1}H_{0m}-H_{0m}^{T}H_{0}^{-1}\frac{\partial H_{0m}}{\partial q_{k}}\\ \;+\;H_{0m}^{T}H_{0}^{-1}\frac{\partial H_{0}}{\partial q_{k}}H_{0}^{-1}H_{0m},\;\forall \;\left(1\leq k\leq N\right) \end{array}$$

As for the time derivative above, the computation of the derivative of the generalized inertia matrix requires the separate computations shown in the following paragraphs.

#### Joint-angle derivative of the manipulator inertia matrix

The joint-angle derivatives of the manipulator inertia matrix from Equation (36) are given by:

(82) $$\begin{array}{l} \frac{\partial H_{m}}{\partial q_{k}}=\sum {\;}_{i=1}^{N}({\frac{\partial J_{Ri}}{\partial q_{k}}}^{T}{\;}^{J}I_{i}J_{Ri}+J_{Ri}^{T}\frac{\partial^{J}I_{i}}{\partial q_{k}}J_{Ri}+J_{Ri}^{T}{\;}^{J}I_{i}\frac{\partial J_{Ri}}{\partial q_{k}}\\ \;+\;m_{i}{\frac{\partial J_{Ti}}{\partial q_{k}}}^{T}J_{Ti}+m_{i}J_{Ti}{\;}^{T}\frac{\partial J_{Ti}}{\partial q_{k}}),\;\forall \;\left(1\leq k\leq N\right) \end{array}$$

The calculation of this expression requires the joint-angle derivatives of the joint and link transformation matrices ${\;}^{J}T_{J_{i}}$ and ${\;}^{J}T_{L_{i}}$, as well as the joint angle derivatives of the linear and angular motion Jacobians. From Equation (9), the joint transformation matrices are:

(83) $$\begin{array}{l} {\;}^{J}T_{J}{\;}_{i}{=}^{J}T_{J}{\;}_{k}•A\left(q_{k},d_{k},\alpha_{k}{,}^{k}a_{k}{+}^{k}b_{k}\right)\\ \;•\prod_{l=k+1}^{i-1}A\left(q_{l},d_{l},\alpha_{l}{,}^{l}a_{l}{+}^{l}b_{l}\right). \end{array}$$

where the homogeneous transformation matrix ${\;}^{J}T_{J}{\;}_{k}$ and the DH matrices *A*($q_{l},d_{l},\alpha_{l}{,}^{l}a_{l}{+}^{l}b_{l}$) (*k* + 1 ≤ *l* ≤ *i* − 1) are independent of joint angle *q*_*k*_. Therefore, only a single DH transformation matrix in the multiplication chain depends on *q*_*k*_, with the joint angle derivative of a DH matrix given by:

(84) $$\begin{array}{l} \frac{\partial A\left(q_{k},d_{k},\alpha_{k}{,}^{k}c_{k}\right)}{\partial q_{k}}=\\ \;\left[\begin{array}{cccc} -\text{ sin }q_{k} & -\;\text{ cos }q_{k}\text{ cos }\alpha_{k} & \text{ cos }q_{k}\text{ sin }\alpha_{k} & -c_{k}\text{ sin }q_{k}\\ \text{ cos }q_{k} & -\;\text{ sin }q_{k}\text{ cos }\alpha_{k} & \text{ sin }q_{k}\text{ sin }\alpha_{k} & c_{k}\text{ cos }q_{k}\\ 0 & 0 & 0 & 0\\ 0 & 0 & 0 & 0 \end{array}\right]. \end{array}$$

Therefore, three cases must be considered:

(1) If *k* > *i* − 1, the joint transformation matrix is independent of *q*_*k*_:

(85) $${\frac{\partial^{J}T_{J}{\;}_{i}}{\partial q_{k}}|}_{k>i-1}=0_{4,4},$$

(2) If *k* = *i* − 1, the final factor in the product of transformation matrices depends on *q*_*k*_, and the derivative changes to:

(86) $${\frac{\partial^{J}T_{J}{\;}_{i}}{\partial q_{k}}|}_{k=i-1}{=}^{J}T_{J}{\;}_{i-1}•\frac{\partial A\left(q_{i-1},d_{i-1},\alpha_{i-1}{,}^{i-1}a_{i-1}{+}^{i-1}b_{i-1}\right)}{\partial q_{i-1}},$$

(3) For *k* < *i* − 1, one of the contributing matrices depends on *q*_*k*_:

(87) $$\begin{array}{l} {\frac{\partial^{J}T_{J}{\;}_{i}}{\partial q_{k}}|}_{k<i-1}{=}^{J}T_{J}{\;}_{k}•\frac{\partial A\left(q_{k},d_{k},\alpha_{k}{,}^{k}a_{k}{+}^{k}b_{k}\right)}{\partial q_{k}}\\ \;•\;\prod_{l=k+1}^{i-1}A\left(q_{l},d_{l},\alpha_{l}{,}^{l}a_{l}{+}^{l}b_{l}\right). \end{array}$$

With (10), the joint angle derivatives of the link transformation matrices become:

(88) $$\;\frac{\partial^{J}T_{L}{\;}_{i}}{\partial q_{k}}\;=\;\frac{\partial^{J}T_{J}{\;}_{i}}{\partial q_{k}}A\left(q_{i},d_{i},\alpha_{i}{,}^{i}a_{i}\right)+{\;}^{J}T_{J}{\;}_{i}\frac{\partial A\left(q_{i},d_{i},\alpha_{i}{,}^{i}a_{i}\right)}{\partial q_{k}}.$$

As for the link transformation matrices ${\;}^{J}T_{L}{\;}_{i}$, there are three cases to consider:

(1) For *k* > *i*, the link transformation matrix is independent of *q*_*k*_.

(89) $${\;\frac{\partial^{J}T_{L}{\;}_{i}}{\partial q_{k}}|}_{k>i}=0_{4,4},$$

(2) For *k* = *i*, the final DH transformation matrix must be differentiated for *q*_*k*_:

(90) $${\;\frac{\partial^{J}T_{L}{\;}_{i}}{\partial q_{k}}|}_{k=i}={\;}^{J}T_{J}{\;}_{i}•\frac{\partial A\left(q_{i},d_{i},\alpha_{i}{,}^{i}a_{i}\right)}{\partial q_{i}},$$

(3) If *k* < *i*, we can reuse the derivative of the joint transformation matrix developed above:

(91) $$\;\frac{\partial^{J}T_{L}{\;}_{i}}{\partial q_{k}}\;=\frac{\partial^{J}T_{J}{\;}_{i}}{\partial q_{k}}A\left(q_{i},d_{i},\alpha_{i}{,}^{i}a_{i}\right).$$

The joint-angle derivatives of the transformation matrices contain the derivatives of both the direction-cosine matrix and the position. Therefore, the derivatives of the angular and linear motion Jacobians can now be determined from Equations (35) and (33) as follows:

(92) $$\frac{\partial J_{Ri}}{\partial q_{k}}=\left[\frac{\partial^{J}R_{J_{1}}}{\partial q_{k}}\begin{array}{cccc} \left(\begin{array}{c} 0\\ 0\\ 1 \end{array}\right) & \cdots & \frac{\partial^{J}R_{J_{i}}}{\partial q_{k}}\left(\begin{array}{c} 0\\ 0\\ 1 \end{array}\right) & 0_{3,N-i} \end{array}\right],\;\begin{array}{c} \forall \;\left(1\leq i\leq N\right)\\ \forall \;\left(1\leq k\leq N\right), \end{array}$$

(93) $$\frac{\partial J_{Ti}}{\partial q_{k}}=\left[\begin{array}{ccccc} \frac{\partial j_{T_{i}{\;}_{1}}}{\partial q_{k}} & \frac{\partial j_{T_{i}{\;}_{2}}}{\partial q_{k}} & \cdots & \frac{\partial j_{T_{i}{\;}_{i}}}{\partial q_{k}} & 0_{3,N-i} \end{array}\right],\;\begin{array}{c} \forall \;\left(1\leq i\leq N\right)\\ \forall \;\left(1\leq k\leq N\right). \end{array}$$

where:

(94) $$\frac{\partial j_{T_{i_{1}}}}{\partial q_{k}}={\left[\frac{\partial^{J}R_{J_{1}}}{\partial q_{k}}\left(\begin{array}{c} 0\\ 0\\ 1 \end{array}\right)\right]}^{\times }\left(r_{i}-p_{l}\right)+{\hat{k}}_{l}^{\times }\left(\frac{\partial r_{i}}{\partial q_{k}}-\frac{\partial p_{l}}{\partial q_{k}}\right),\;\begin{array}{c} \forall \;\left(l\leq i\right)\\ \forall \;\left(1\leq k\leq N\right). \end{array}$$

#### Joint-angle derivative of the dynamic-coupling matrix

The derivatives of the dynamic-coupling matrix are computed from Equation (31) as:

(95) $$\frac{\partial H_{0m}}{\partial q_{k}}=\left[\begin{array}{c} \frac{\partial J_{TS}}{\partial q_{k}}\\ \frac{\partial H_{Sq}}{\partial q_{k}} \end{array}\right],\;\forall \;(1\leq k\leq N),$$

where

(96) $$\frac{\partial J_{TS}}{\partial q_{k}}=\sum_{i=1}^{N}\left(m_{i}\frac{\partial J_{Ti}}{\partial q_{k}}\right),\;\forall \;\left(1\leq k\leq N\right),$$

(97) $$\begin{array}{l} \frac{\partial H_{Sq}}{\partial q_{k}}=\sum_{i=1}^{N}\left\{\frac{\partial^{J}I_{i}}{\partial q_{k}}J_{Ri}{+}^{J}I_{i}\frac{\partial J_{Ri}}{\partial q_{k}}+m_{i}\left[{\left(\frac{\partial^{J}r_{0i}}{\partial q_{k}}\right)}^{\times }J_{Ti}+r_{0i}{\;}^{\times }\frac{\partial J_{Ti}}{\partial q_{k}}\right]\right\},\\ \;\forall \;\left(1\leq k\leq N\right). \end{array}$$

Since ***r***_**0**_ does not depend directly on any joint angle, ***r***_**0*i***_ (see Equation 11) is differentiated as:

(98) $$\frac{\partial r_{0i}}{\partial q_{k}}=\frac{\partial r_{i}}{\partial q_{k}},\;\begin{array}{c} \forall \;\left(1\leq i\leq N\right)\\ \;\forall \;\left(1\leq k\leq N\right). \end{array}$$

#### Joint-angle derivative of the base-spacecraft inertia matrix

The joint-angle derivatives of the base-spacecraft inertia matrix ***H***_**0**_ from Equation (28) result in:

(99) $$\frac{\partial H_{0}}{\partial q_{k}}=\left[\begin{array}{cc} 0_{3,3} & -m_{tot}{\left(\frac{\partial r_{0C}}{\partial q_{k}}\right)}^{\times }\\ m_{tot}{\left(\frac{\partial r_{0C}}{\partial q_{k}}\right)}^{\times } & \frac{\partial H_{S}}{\partial q_{k}} \end{array}\right].$$

The joint-angle derivatives of the relative center-of-mass position vector ***r***_**0*C***_ from Equation (13) are calculated in:

(100) $$\begin{array}{l} \frac{\partial r_{0C}}{\partial q_{k}}=\frac{1}{m_{tot}}\sum_{i=1}^{N}\left(m_{i}\frac{\partial r_{0i}}{\partial q_{k}}\right)=\frac{1}{m_{tot}}\sum_{i=1}^{N}\left(m_{i}\frac{\partial r_{i}}{\partial q_{k}}\right),\\ \;\forall \;\left(1\leq k\leq N\right). \end{array}$$

The system moments-of-inertia matrix from Equation (29) is differentiated by:

(101) $$\begin{array}{l} \frac{\partial H_{S}}{\partial q_{k}}=\sum_{i=1}^{N}\left\{\frac{\partial^{J}I_{i}}{\partial q_{k}}-m_{i}\left[{\left(\frac{\partial r_{i}}{\partial q_{k}}\right)}^{\times }r_{0i}{\;}^{\times }+r_{0i}{\;}^{\times }{\left(\frac{\partial r_{i}}{\partial q_{k}}\right)}^{\times }\right]\right\},\\ \;\forall \;\left(1\leq k\leq N\right), \end{array}$$

where from Equation (30):

(102) $$\begin{array}{l} \frac{\partial^{J}I_{i}}{\partial q_{k}}=\frac{\partial^{J}R_{L_{i}}}{\partial q_{k}}{\;}^{L_{i}}I_{i}{\;}^{J}R_{L_{i}}^{T}{+}^{J}R_{L_{i}}{\;}^{L_{i}}I_{i}{\frac{\partial^{J}R_{L_{i}}}{\partial q_{k}}}^{T}\\ \;\forall \;\left(1\leq k\leq N\right). \end{array}$$

## Generalized Jacobian

For control purposes, a transformation between joint-space and task-space requires the use of a Jacobian (Siciliano et al., 2010). Typically, the Jacobian is used to map generalized joint velocities to a spatial velocity of the end-effector. However, for reasons of collision avoidance and contact dynamics, Jacobians must also be expressed for the combined velocity of any arbitrary point *X*_*i*_ on the *i*-th link of the manipulator. From Equations (15) and (16), it follows:

(103) $$\left[\begin{array}{c} \nu_{X_{i}}\\ \omega_{i} \end{array}\right]=J_{0_{X_{i}}}{\dot{x}}_{0}+J_{m_{X_{i}}}\dot{q},$$

where the contribution of the manipulator joint rates to the combined velocity of *X*_*i*_ is expressed by the [6 × *N*] manipulator Jacobian ***J***_***m***_***X***_***i***___:

(104) $$J_{m_{X_{i}}}=\left[\begin{array}{cccc} {\hat{k}}_{1}^{\times }\left(x_{i}-p_{1}\right) & \ldots & {\hat{k}}_{i}^{\times }\left(x_{i}-p_{i}\right) & 0_{3,N-i}\\ {\hat{k}}_{1} & \ldots & {\hat{k}}_{i} & 0_{3,N-i} \end{array}\right],$$

with ***x***_***i***_ being the position of point *X*_*i*_. The contribution of the base-spacecraft combined velocity is expressed by the [6 × 6] base-spacecraft Jacobian ***J***_**0**_***X***_***i***___:

(105) $$J_{0_{X_{i}}}=\left[\begin{array}{cc} E & -x_{0i}^{\times }\\ 0_{3,3} & E \end{array}\right],$$

where

(106) $$x_{0i}=x_{i}-p_{0}.$$

When using the generalized form of the equations of motion, a [6 × *N*] generalized Jacobian $J_{X_{i}}^{⋆}$ can be formulated, such that:

(107) $$\left[\begin{array}{c} {\;}^{J}\nu_{X_{i}}\\ {\;}^{J}\omega_{i} \end{array}\right]=J_{X_{i}}^{⋆}\dot{q}.$$

Therefore:

(108) $$J_{X_{i}}^{⋆}\dot{q}=J_{0_{X_{i}}}{\dot{x}}_{0}+J_{m_{X_{i}}}\dot{q}.$$

Using Equation (42) and assuming ***M***_**0**_ = **0**_**6, 1**_, ${\dot{x}}_{0}$ can be expressed as:

(109) $${\dot{x}}_{0}=-H_{0}^{-1}H_{0m}\dot{q}.$$

The generalized Jacobian is thus defined as:

(110) $$J_{X_{i}}*=J_{m_{X_{i}}}-J_{0_{X}{\;}_{i}}H_{0}^{-1}H_{0m}$$

If ***x**_**i**_* coincides with the position of the end-effector (***p***_***E***_ = ***p***_***N***+**1**_), the resulting generalized Jacobian Jacobian defined by Equation (110), for an arbitrary point, becomes the same as the Generalized Jacobian ***J***^⋆^ of the floating manipulator as originally defined by Yoshida and Umetani (1993). Therefore, the joint rates required to have the end-effector move at a linear velocity **ν**_***E***_ and angular velocity **ω**_***E***_ can be calculated from:

(111) $$\dot{q}=J^{{⋆}^{-1}}\left[\begin{array}{c} \nu_{E}\\ \omega_{E} \end{array}\right]$$

## Implementation of a simulation model for a floating spacecraft-manipulator system

**Figure 3:**
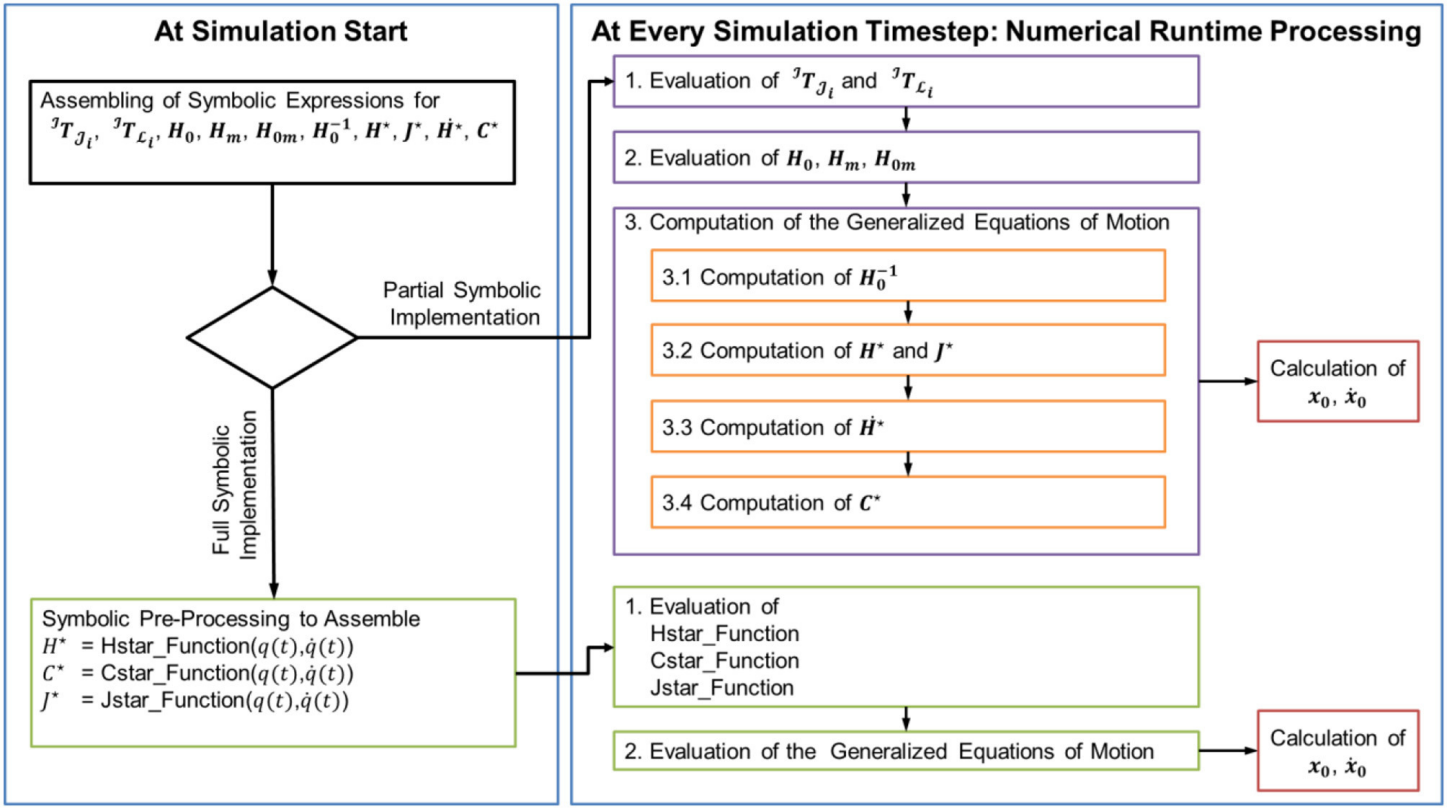
Pseudo-code structure illustrating the two proposed implementation variants of a floating spacecraft-manipulator system simulation model based on the Generalized Jacobian approach.

By using the generalized equations in symbolic form introduced above, a computer model can be implemented for the simulation of the floating spacecraft-manipulator system. This computer model can be based on two implementation approaches, which we call here *full symbolic implementation* and *partial symbolic implementation* (see Figure 3). The numerical simulation can thus be run by evaluating at each integration step static functions containing symbolic expressions, without executing iterative procedures such as the recursive Newton-Euler algorithm as in the available literature on the subject (refer to, for instance Mukherjee and Nakamura, 1992; Carignan and Akin, 2000). In the *partial symbolic implementation*, the numerical values for matrices ${\;}^{J}T_{J}{\;}_{i}$, ${\;}^{J}T_{L}{\;}_{i}$, ***H***_0_, ***H*_*0m*_**, ***H***_***m***_, and ${\text{H}}_{0}^{\text{-1}}$ are calculated from their symbolic expressions. These numerical values are then used to find the generalized matrices ***H***^⋆^, ***C***^⋆^, and ***J***^⋆^. In the full symbolic implementation, ***H***^⋆^, ***C***^⋆^, ***J***^⋆^ are calculated directly from their symbolic expressions.

The symbolic implementations are easily adaptable to any number of joints and any structure of the kinematic chain of the spacecraft-manipulator system.

## Numerical simulations

**Figure 4:**
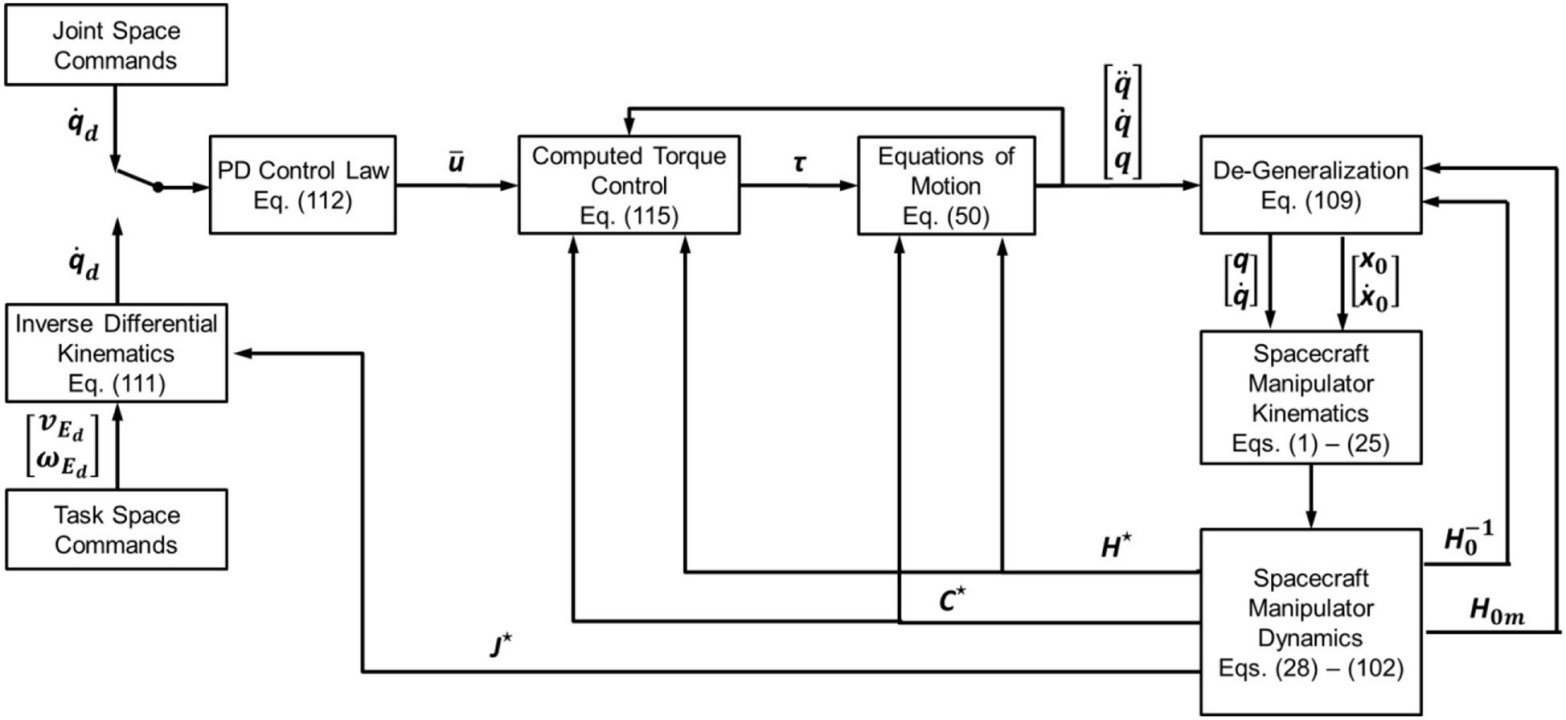
Block diagram of the overall simulated control architecture used for the numerical simulations.

In order to verify the proposed analytical approach, a simulation model was implemented for two case studies: (1) a planar spacecraft-manipulator system with a 4 DOF manipulator, as commonly used in ground experimentation of spacecraft-manipulator systems, and (2) a spatial spacecraft-manipulator system with a spatial 6 DOF manipulator, similar to the one described in Yoshida's original discussion of the generalized Jacobian approach (Yoshida and Umetani, 1993). The simulation model was implemented in Matlab/Simulink by following the partial symbolic implementation approach outlined in Figure 3. The block diagram of the overall simulated control architecture is illustrated in Figure 4.

With the complete knowledge of the generalized equations of motion of the spacecraft-manipulator system, the system can be controlled using a standard control scheme called Computed Torque Control (Siciliano et al., 2010). In particular, the commanded torque for the joint motors is computed from the desired joint angular acceleration, angular rate, and angular position by means of a direct dynamics operation. The joint acceleration control input $\bar{u}$ is computed by means of a proportional-derivative (PD) control law:

(112) $$\bar{u}=K_{D}\left({\dot{q}}_{D}-\dot{q}\right)+K_{P}\left(q_{D}-q\right)$$

In general, the controller gains can be designed based on the solutions of the harmonic oscillator:

(113) $$K_{P_{i}}=\frac{\tau_{max,i\;}}{{\left(q_{D}-q\right)}_{max}},$$

(114) $$K_{D_{i}}=\sqrt{2}K_{P_{i}},$$

with *T*_*max,i*_ being the maximum motor torque for each joint. The control input is finally fed into the dynamic model as acceleration to derive the resulting reaction torque. This torque then serves as the input to the real manipulator system. With a manipulator employed on an orbiting spacecraft, there is always a substantial level of uncertainty when it comes to the inertial properties of the spacecraft-manipulator system, mostly due to the settling state of the fuel tanks and the associated fuel sloshing. Therefore, the computed torque control is based on estimates of the inertia properties matrices, ${\tilde{H}}^{⋆}$ and ${\tilde{\text{C}}}^{⋆}$:

(115) $$\tau ={\tilde{H}}^{⋆}\bar{u}+{\tilde{C}}^{⋆}(q,q\dot{\;}).$$

In the numerical simulation presented here, perfect knowledge of the inertia properties of the system was assumed, thus ${\tilde{\text{H}}}^{⋆}={\text{H}}^{⋆}$ and ${\tilde{\text{C}}}^{⋆}={\text{C}}^{⋆}$.

### Simulations with the planar four-link spacecraft-manipulator system

The simulated planar spacecraft-manipulator system consists of a base-spacecraft and four identical manipulator links. The mass and inertia properties of the system are given in Table 3, whereas the properties of the kinematic chain are summarized in Table 4. For illustration purposes, the gains of the controller were set to *K*_*D*_*i*__ = 1 and *K*_*P*_*i*__ = 1 for all joints *i*. The manipulator is initially fully extended along the x axis, which corresponds to zero angle for each joint. In the sample maneuver sequence one joint at a time accelerates to 0.1 rad/s for 10 s, then rests for 2 s, rotates at −0.1 rad/s for 20 s, rests again for 2 s, then rotates back to the initial condition. The base-spacecraft is initially located at the origin of the inertial coordinate system, with the three Euler angles being all zero.

**Table 3:** Planar spacecraft-manipulator system with four-link manipulator: mass and inertia properties.

**Link**	**Mass [kg]**	**Length [m]**	**Width [m]**	**Height [m]**	**Principal Moments-of-inertia [kg m**^**2**^**]**		
					***I*_*xx*_**	***I*_*yy*_**	***I*_*zz*_**
Base-spacecraft	10	0.2	0.2	0.8	0.5667	0.5667	0.0667
Links 1–4	2	0.4	0.08	0.18	0.0065	0.0321	0.0277

**Table 4:** Planar spacecraft-manipulator system with four-link manipulator: customized Denavit-Hartenberg parameters.

**Joint**	**Denavit-Hartenberg parameters**			
	***d*[m]**	**α [°]**	***a*[m]**	***b*[m]**
2	0	0	0.2	0.2
3	0	0	0.2	0.2
4	0	0	0.2	0.2
End-effector	0	0	0.2	0.2

**Figure 5:**
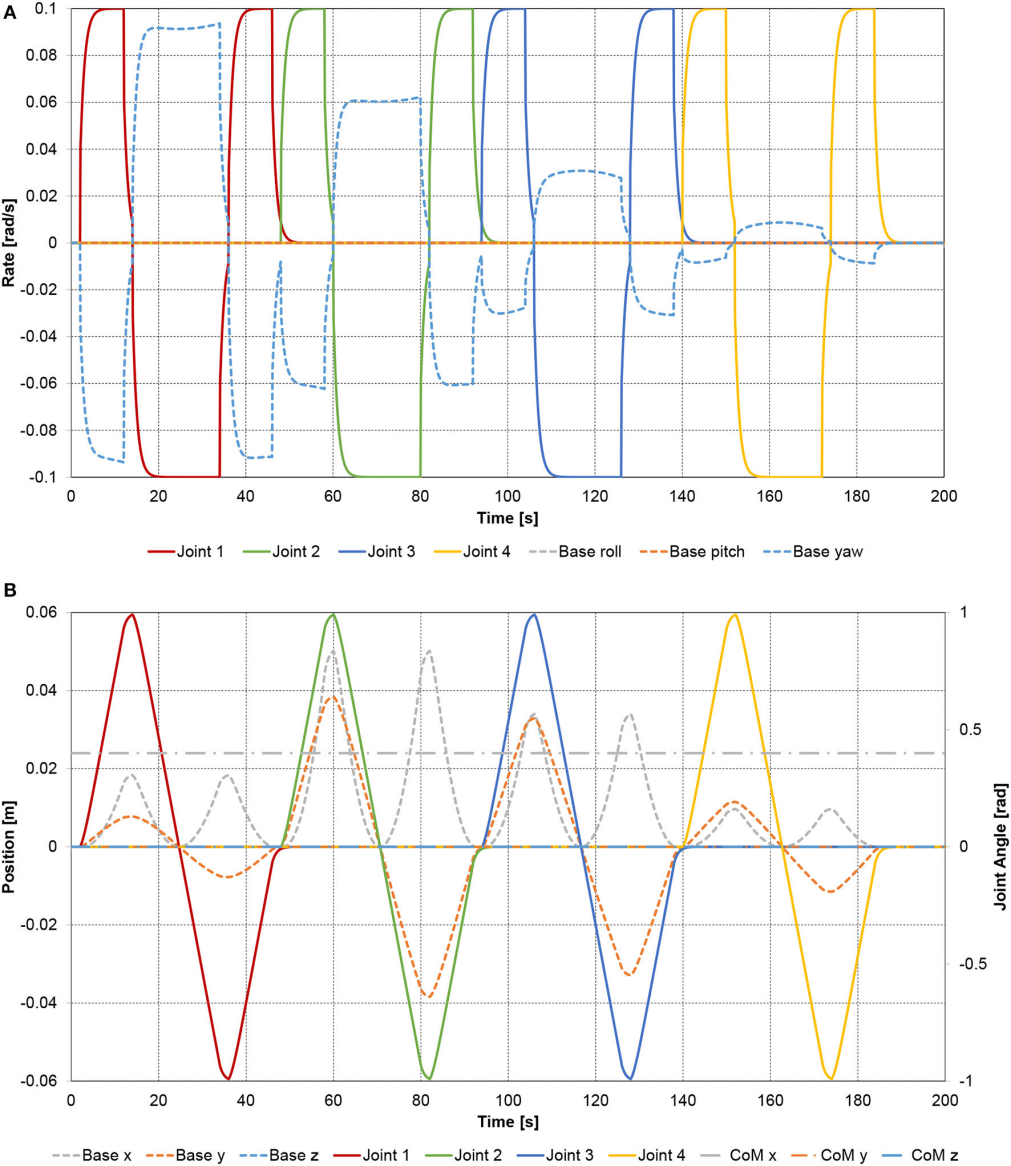
Planar spacecraft-manipulator system with four-link manipulator: **(A)** joint rates and the resulting base-spacecraft angular velocity components; **(B)** joint angles and the resulting base-spacecraft position deviation.

As shown in Figure 5A, the dynamic coupling between the base-spacecraft and the manipulator generates compensatory motion of the base-spacecraft. As expected, the base-spacecraft has an angular rate component which has opposite sign with respect to the joint rates, and is smaller in magnitude, since the base-spacecraft has higher inertia than the manipulator. Of note is also that the base-spacecraft reaction becomes smaller as the joint maneuver sequence proceeds “outward”, since the outer joints move less mass than the inner joints.

Similar behavior is evident in Figure 5B, which illustrates the base-spacecraft position deviation depending on the joint angles. Since the total center-of-mass of the system remains stationary, the base-spacecraft center-of-mass is pulled forward and pushed back to its initial *x* position, and is pushed to the left and right in the *y* direction as the manipulator moves to the left and right. The magnitude of the position deviation depends on the combination of distance between base-spacecraft center-of-mass and the center-of-mass of the actuated links, and the total mass or the actuated links. The location of the total center of mass of the multi-body system remains constant.

**Figure 6:**
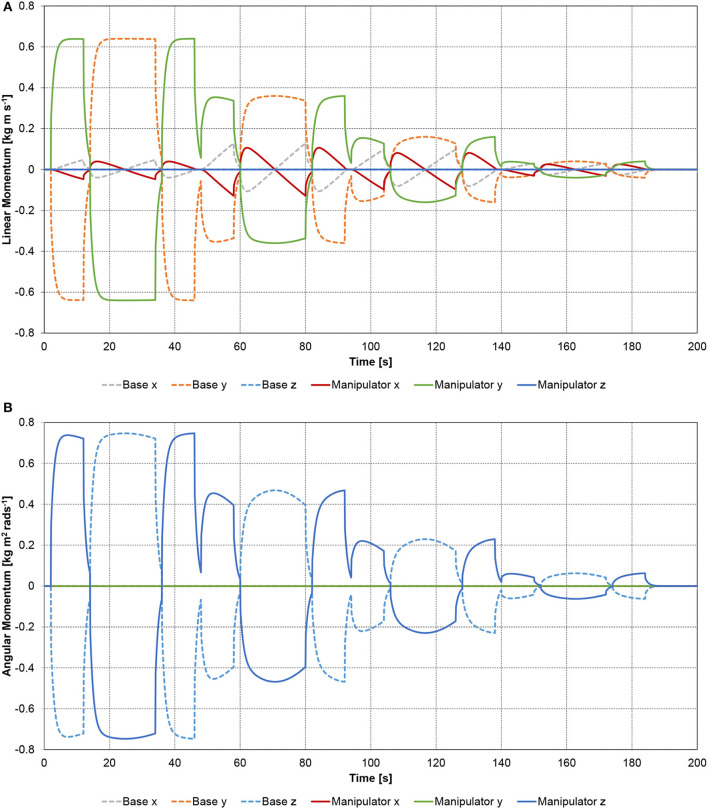
Planar spacecraft-manipulator system with four-link manipulator: **(A)** linear momentum; **(B)** angular momentum.

Since the manipulator joints are in this example purely actuated by internal torques, both the linear momentum and the angular momentum of the spacecraft-manipulator system remain constant, and in this case zero, as expected. Therefore, the linear and angular momenta of the base-spacecraft must be opposite in sign but equal in magnitude to the manipulator momenta. This expected behavior is confirmed in Figures 6A,B. Since the spacecraft-manipulator system is only moving in the x-y plane, there is no linear momentum along the z axis, and no angular momentum about the x and y axes.

### Simulations with the spatial six-link spacecraft-manipulator system

The simulated spatial spacecraft-manipulator system consists of a base-spacecraft and six manipulator links. The mass and inertia properties of the system are given in Table 5, the DH parameters are summarized in Table 6. For illustration purposes, the gains of the controller were set to *K*_*D*_*i*__ = 1 and *K*_*P*_*i*__ = 1 for all joints *i*. The manipulator is initially fully extended along the x axis, which corresponds to zero angle for each joint. In the sample maneuver sequence, one joint at a time accelerates to 0.1 rad/s for 10 s, then rests for 2 s, rotates at −0.1 rad/s for 20 s, rests again for 2 s, then rotates back to zero angle. The base-spacecraft is initially located at the origin of the inertial coordinate system, with the three Euler angles being zero.

**Table 5:** Spatial spacecraft-manipulator system with six-link manipulator: mass and inertia properties (Yoshida and Umetani, 1993).

**Link**	**Mass [kg]**	**Length [m]**	**Principal moments-of-inertia [kg m**^**2**^**]**		
			***I*_*xx*_**	***I*_*yy*_**	***I*_*zz*_**
Base-spacecraft	1,700	3.5	1,434	1,434	1,735
1	5	0.5	0.0292	0.0292	0.0063
2	50	2.5	0.0625	26.1000	26.1000
3	50	2.5	0.0625	26.1000	26.1000
4	10	0.5	0.0125	0.2150	0.2150
5	5	0.25	0.0063	0.0292	0.0292
6	5	0.25	0.0063	0.0292	0.0292

**Table 6:** Spatial spacecraft-manipulator system with six-link manipulator: customized Denavit-Hartenberg parameters.

**Joint**	**Denavit-Hartenberg parameters**			
	***d*[m]**	**α [°]**	***a*[m]**	***b*[m]**
2	0.25	90	0	0
3	0	0	1.25	1.25
4	0	0	1.25	1.25
5	0	−90	0.25	0.25
6	0	0	0.125	0.125
End-effector	0	0	0.125	0.125

**Figure 7:**
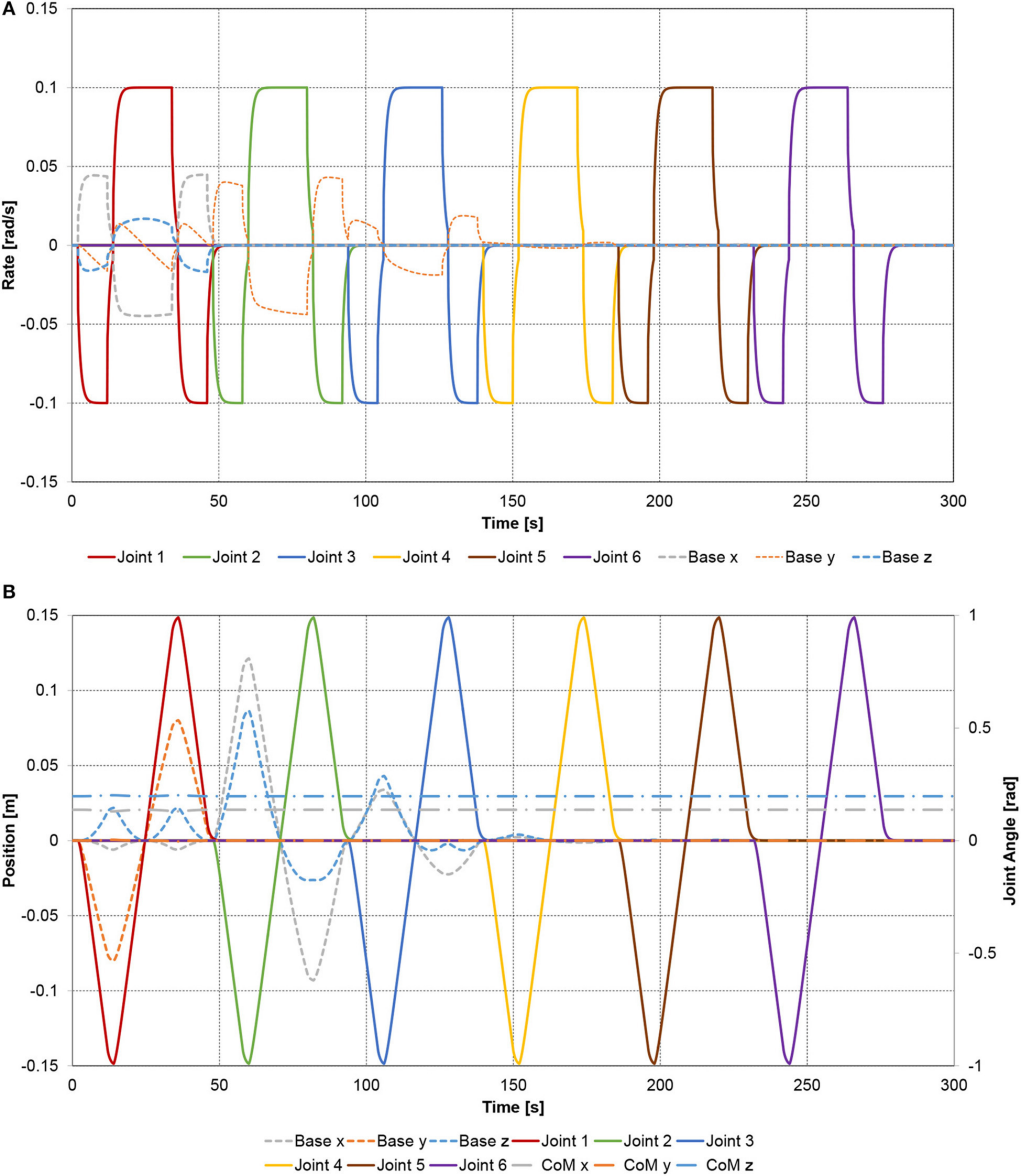
Spatial spacecraft-manipulator system with six-link manipulator: **(A)** joint rates and the resulting base-spacecraft angular velocity components; **(B)** joint angles and the resulting base-spacecraft position deviation.

**Figure 8:**
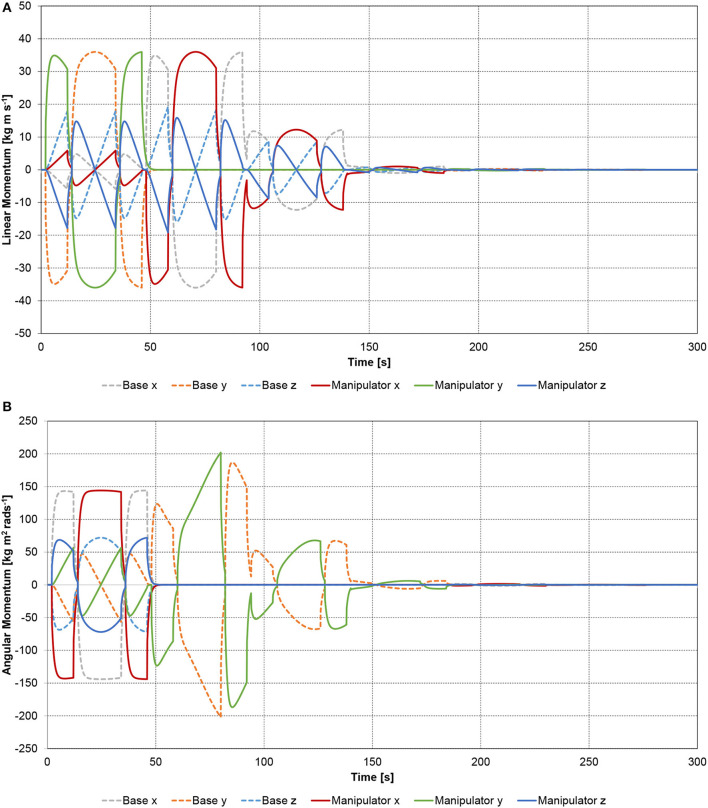
Spatial spacecraft-manipulator system with six-link manipulator: **(A)** linear momentum; **(B)** angular momentum.

The rotation axis of joint 1 is along the *x* axis of the spacecraft coordinate system. Therefore, the rotation of joint 1 causes a 3 DOF rotation of the base-spacecraft, see Figure 7A. Furthermore, it causes the center-of-mass of the base-spacecraft to deviate from its initial position in *x, y* and *z* direction during the joint 1 cycle, and only in *x* and *z* direction when joints 2–6 are active, as shown in Figure 7B. The location of the total center of mass of the multi-body system remains constant. The linear and angular momenta of base-spacecraft and manipulator compensate each other (see Figure 8) as the total momentum is conserved.

## Conclusion

This paper presents a complete, step-by-step analytic derivation of the equations of motion of a floating spacecraft-manipulator system, using the Generalized Jacobian approach for modeling the dynamics of a spacecraft-manipulator system. This includes symbolic analytic expressions for all inertia matrices of the spacecraft-manipulator system, as well as their time derivatives and joint-angle derivatives. It also includes a general expression for the Jacobian of a generic point on any of the links of the spacecraft-manipulator system, which is required for the analysis and simulation of collision-avoidance systems and contact dynamics. Furthermore, new and more detailed definitions are proposed in this paper (see Table 1) for the possible modes of maneuvering a spacecraft-manipulator system. In particular, the two commonly used categories (free-flying and free-floating) are expanded by the introduction of five categories (namely floating, rotation-floating, rotation-flying, translation-flying, and flying). The authors believe that the adoption of the newly proposed definitions would contribute to increased clarity, advantageous to both students and researchers in the field. Furthermore, the paper introduces a full-symbolic implementation and a partial-symbolic implementation of computer simulation models based on the complete generalized Jacobian approach. These implementation options allow the development of efficient numerical simulations. Example simulations, for a planar four-link spacecraft-manipulator system, and a spatial six-link spacecraft manipulator system, show that the modeling approach is effective and consistent with the physical principles.

The description of the geometry of the spacecraft-manipulator system uses DH parameters, which allows complete generality. The approach is thus, in principle, extendable to multiple manipulators with open-chain configuration. A fundamental assumption that was used in the description of the kinematics and dynamics of the spacecraft-manipulator system is that of straight links with the center-of-mass of the link being located on the straight line through the origins of the adjacent Cartesian joint coordinate systems. While this is a restriction of generality, it also reflects the type of robotic manipulators commonly used in orbital robotics systems. Furthermore, only rotary joints were considered, since prismatic joints are not commonly used in orbital robotics applications. However, the description of the kinematics and dynamics of the system can be, in principle, straightforwardly extended to include both irregularly shaped links and prismatic joints. Gears in the joints were not considered but they could be added in the analysis by using, for instance, the approach detailed in Siciliano et al. (2010). Euler angles were used in the description of the base-spacecraft orientation: in principle it would be straightforward to use quaternions to avoid any possible orientation singularity problem.

The goal is for this detailed presentation of the Generalized Jacobian approach to serve as a tutorial to build a complete analytic model of the complex dynamics of a spacecraft-manipulator system. While this tutorial is mostly aimed at aerospace engineers faced with the challenge of modeling a robotic system while designing a spacecraft for a rendezvous and capture mission, it is also a good reference for robotic engineers, since it condenses material from distributed sources into one complete presentation.

## Author contributions

MW is the principal author. He performed the literature research into the generalized Jacobian approach and derived the mathematical equations to fill the gaps in published literature. He also implemented the Matlab/Simulink model used to generate the examples shown in the paper. He also had the lead in writing the paper. SK worked with MW on checking the mathematical derivations for correctness and on clarifying the presentation of the Denavit-Hartenberg convention. AG worked with MW on the implementation in Matlab/Simulink and on producing the data plots used in the paper. MR provided expertise on the Lagrangian method used to derive the equations of motion and on matrix/vector mathematics.

### Conflict of interest statement

The authors declare that the research was conducted in the absence of any commercial or financial relationships that could be construed as a potential conflict of interest.

## Nomenclature

J = Inertial Cartesian coordinate system orbiting with the spacecraft-manipulator system

$J_{i}$ = Cartesian coordinate system attached to joint *i*

$L_{i}$ = Cartesian coordinate system attached to link *i*

α_*i*_ = Denavit-Hartenberg parameter for rotation from link coordinate system axis *z*_*i−1*_ to axis *z*_*i*_ about axis *x*_*i*_

**ω**_***E***_ = angular velocity of the end-effector with respect to inertial frame J

**ω**_***i***_ = angular velocity of link *i* with respect to inertial frame J

**ω**_0_ = base-spacecraft angular velocity with respect to inertial frame J

Ω = origin of the inertial Cartesian coordinate system J

${\left[\begin{array}{c} \epsilon \;\&\zeta \&\;\eta \end{array}\right]}^{T}$ = Euler angles (sequence 1-2-3) expressing the orientation of the coordinate system attached to joint 1 with respect to the coordinate system at the spacecraft center-of-mass CM_0_

${\left[\begin{array}{c} \phi \;\&\theta \&\Psi \end{array}\right]}^{T}$ = Euler angles (sequence 1-2-3) expressing the orientation of the base-spacecraft (link 0) with respect to the inertial coordinate system J

ϱ_*i*_ = origin of the Cartesian coordinate system attached to joint *i*

**τ** = [*N* × 1] matrix of joint torques (each element τ_*i*_ expresses the scalar value of the torque component along ${\hat{k}}_{i}$ due to the motor driving joint *i*)

θ_*i*_ = Denavit-Hartenberg parameter for rotation between link coordinate system axis *x*_*i−1*_ and *x*_*i*_ about axis *z*_*i−1*_

${\;}_{a}^{ℬ}$ = [3 × 1] matrix of components of the vector ***a*** along the Cartesian coordinate system B

***a*** ^×^ = [3 × 3] skew-symmetric matrix associated with vector ***a***

***a***_*i*_ = position vector from ϱ_*i*_ to center-of-mass of link *i*

***A*** = [4 × 4] Denavit-Hartenberg transformation matrix

***b*_0_** = position vector from CM_0_ to ϱ_1_

***b*_*i*_** = position vector from CM_i_ to ϱ_*i*+1_

*c*_*i*_ = Denavit-Hartenberg parameter for displacement between link coordinate system axis *z*_*i−1*_ and axis *z*_*i*_ along axis *x*_*i*_

***c***^⋆^ = [*N* × 1] matrix of generalized non-linear terms of the equations of motion

***C***^⋆^ = [*N* × 1] matrix of generalized centripetal and Coriolis terms

${\tilde{C}}^{⋆}$ = [*N* × 1] matrix of estimated generalized centripetal and Coriolis terms

***c*_*0*_** = [6 × 1] matrix of derivatives for base-spacecraft position

***c*_*m*_** = [*N* × 1] matrix of derivatives for manipulator joint angles

CM_i_ = center-of-mass of link *i*

CM_tot_ = spacecraft-manipulator system center-of-mass

*d*_*i*_ = Denavit-Hartenberg parameter for displacement between link coordinate system axis *x*_*i−1*_ and axis *x*_*i*_ along axis *z*_*i−1*_

**ε** = [3 × 1] vector part of unit quaternion ***q***

η = Scalar part of unit quaternion ***q***

𝕀_3, 3_ = [3 × 3] identity matrix

***H*^⋆^** = [*N* × *N*] generalized inertia properties matrix

${\tilde{H}}^{⋆}$ = [*N* × *N*] estimated generalized inertia properties matrix

***H*_0_** = [6 × 6] base inertia properties matrix

***H*_0*m*_** = [6 × *N*] base/manipulator dynamic-coupling matrix

***H*_*m*_** = [*N* × *N*] manipulator inertia properties matrix

***H*_*S*_** = [3 × 3] system moments-of-inertia matrix

***H*_*Sq*_** = [3 × *N*] angular motion energy contribution matrix

ILi i = [3 × 3] moments-of-inertia matrix of link *i* assembly with respect to the Cartesian coordinate system attached to link *i*

${\;}^{J}I_{i}$ = [3 × 3] moments-of-inertia matrix of link *i* assembly with respect to the inertial coordinate system J

***J***^⋆^ = [6 × *N*] generalized Jacobian for the motion of the end-effector

$J_{X_{i}}^{⋆}$ = [6 × *N*] generalized Jacobian for the motion of *X*_*i*_

***J*_*0*_*X*_*i*___** = [6 × 6] Jacobian for the contribution of the base-spacecraft motion to the velocity of *X*_*i*_

$J_{k}^{⋆}$ = [6 × *N*] generalized Jacobian for point of action of external force or torque ***F*_*k*_**

***J*_*m*_*X*_*i*___** = [6 × *N*] Jacobian for the contribution of the manipulator motion to the velocity of *X*_*i*_

***J*_*Ri*_** = [3 × *N*] angular motion Jacobian for the center-of-mass of link *i*

***J*_*Ti*_** = [3 × *N*] linear motion Jacobian for the center-of-mass of link *i*

***j*_*T*_*i*__*k*__** = [3 × 1] k-th column of matrix ***J*_*Ti*_**

***J*_*TS*_** = [3 × *N*] linear motion energy contribution matrix

***K*_*D*_** = [*N* × 1] matrix of elements *K*_*D*_*i*__

***K*_*D*_*i*__** = derivative controller gain

${\hat{k}}_{i}$ = unit vector along the z axis of the coordinate system attached to joint *i*

***K*_*P*_** = [*N* × 1] matrix of elements *K*_*P*_*i*__

*K*_*P*_*i*__ = proportional controller gain

*L* = Lagrangian

***L*** = angular momentum vector about CM_0_ expressed in frame J

***m*_*i*_** = mass of link *i* assembly (including connected parts of joints *i* and *i+1*)

${\;}^{J}M_{0}$ = [6 × 1] combined matrix of initial momentum (linear and angular) with respect to the base-spacecraft center-of-mass

*m*_*tot*_ = mass of the spacecraft-manipulator system

*N* = number of manipulator joints

***0*_*i, j*_** = [*i* × *j*] zero matrix

***P*** = linear momentum vector about CM_0_ expressed in frame J

***p*_*i*_** = position vector from point Ω to the point ϱ_*i*_

***q*** = [*N* × 1] matrix of elements *q*_*i*_

***q*_*D*_** = [*N* × 1] matrix of elements *q*_*D*_

***q*** = [4 × 1] unit quaternion

*q*_*D*_*i*__ = desired angular displacement of joint *i*

*q*_*i*_ = angular displacement of joint *i*

qB rA = [4 × 1] unit quaternion expressing the rotation between coordinate system A and coordinate system B

***r*_*C*_** = position vector from Ω to *CM*_*tot*_ expressed in frame J

***r*_*i*_** = position vector from point Ω to point *CM*_*i*_ expressed in frame J

***r*_*0C*_** = position vector from *CM*_*0*_ to *CM*_*tot*_ expressed in frame J

***r*_*0i*_** = position vector from *CM*_0_ to *CM*_*i*_ expressed in frame J

RB A = [3 × 3] direction-cosine matrix from the coordinate system A to the coordinate system B

***S*_*U*_** = [3 × 3] Schur complement matrix of upper left sub-matrix of ***H*_*b*_**

*s*_*j, k*_ = row *j*, column *k* element of ***S*_*U*_**

tB A = [4 × 1] four-vector expressing the translation of the coordinate system origin between system A and system B

*T* = kinetic energy

**τ**_*max, i*_ = maximum motor torque for joint *i*

TB A = [4 × 4] homogenous transformation matrix from the coordinate system A to the coordinate system B

***u*** = [*N* × 1] matrix of control inputs for Computed Torque Control

***v*_0_** = linear velocity of the base-spacecraft with respect to the inertial frame J

***v*_*E*_** = linear velocity of the end-effector with respect to the inertial frame J

***v*_*X*_*i*__** = linear velocity of arbitrary point *X*_i_ located on link *i* of the manipulator with respect to the inertial frame J

${\;}^{J}x_{0}$ = [6 × 1] combined position matrix of linear position components and attitude parameters of the base-spacecraft, (${\;}^{J}x_{b}$ = ${\left[\begin{array}{cccc} {\;}^{J}r_{0}{\;}^{T} & \phi & \theta & \psi \end{array}\right]}^{T}$)

${\;}^{J}{\dot{x}}_{0}$ = [6 × 1] combined velocity matrix of linear velocity components and angular velocity components of the base-spacecraft,

$$\begin{array}{l} {(}^{J}{\dot{x}}_{0}={\left[\begin{array}{cc} {\;}^{J}v_{0}{\;}^{T} & {\;}^{J}\omega_{0}{\;}^{T} \end{array}\right]}^{T}={\left[\begin{array}{cc} {\;}^{J}v_{0}{\;}^{T} & {\left(\left[\begin{array}{ccc} 1 & 0 & -\text{ sin }\theta \\ 0 & \text{ cos }\phi & \text{ cos }\theta \text{ sin }\phi \\ 0 & -\text{ sin }\phi & \text{ cos }\phi \text{ cos }\theta \end{array}\right]\left[\begin{array}{c} \dot{\phi }\\ \dot{\theta }\\ \dot{\psi } \end{array}\right]\right)}^{T} \end{array}\right]}^{T}) \end{array}$$

***x*_*0i*_** = position vector from CM_0_ to an arbitrary point on link *i*

*X*_*i*_ = arbitrary point on link *i*

***x*_*i*_** = position vector from Ω to an arbitrary point on link *i*

DCM = Direction-Cosine Matrix

DEM = Dynamically Equivalent Manipulator

DH = Denavit-Hartenberg

DOF = Degree of Freedom

EE = End-Effector

ETS-VII = Engineering Test Satellite VII

GJM = Generalized Jacobian Matrix

HTV = H-II Transfer Vehicle

ISS = International Space Station

JEMRMS = Japanese Experiment Module Remote Manipulator System

PD = proportional-derivative controller

SPDM = Special Purpose Dexterous Manipulator

SRMS = Shuttle Remote Manipulator System

VM = Virtual Manipulato.

## References

[B1] Aghili F. (2009). Coordination control of a free-flying manipulator and its base attitude to capture and detumble a cooperative satellite, in Proceedings of 2009 IEEE/RSJ International Conference on Intelligent Robots and Systems (St. Louis, MO), 2365–2372.

[B2] Bain M. E. (2010). Cygnus: back to the future - applying commercial program lessons learned, in AIAA SPACE 2010 Conference & Exposition (Anaheim, CA: AIAA), 1–8.

[B3] Baksalary J. K. Styan G. P. H. (2002). Generalized inverses of partitioned matrices in Banachiewicz-Schur form. Linear Algeb. Appl. 354, 41–47. 10.1016/S0024-3795(02)00334-8

[B4] Barnhart D. Sullivan B. Hunter R. Bruhn J. Fowler E. Hoag L. (2013). Phoenix project status 2013, in AIAA Space 2012 Conference and Exhibition (San Diego, CA: AIAA), 1–17.

[B5] Caccavale F. Siciliano B. (2001). Quaternion-based kinematic control of redundant spacecraft/manipulator systems, in Proceedings of the 2001 IEEE International Conference on Robotics and Automation (Seoul: IEEE), 435–440.

[B6] Campa R. Camarillo K. (2008). Unit quaternions: a mathematical tool for modeling, path planning and control of robot manipulators, in Robot Manipulators, ed Ceccarelli M. (Rijeka: InTech), 23–48.

[B7] Carignan C. R. Akin D. L. (2000). The reaction stabilization of on-orbit robots. IEEE Control Syst. Mag. 20, 19–33. 10.1109/37.887446

[B8] Cepollina F. (2013). Robotic Refueling Mission. NASA (Accessed January 6, 2014). Available online at: http://www.nasa.gov/mission_pages/station/research/experiments/778.html.

[B9] Coleshill E. Oshinowo L. Rembala R. Bina B. Daniel R. Shelley S. (2009). Dextre: improving maintenance operations on the International Space Station. Acta Astronaut. 64, 869–874. 10.1016/j.actaastro.2008.11.011

[B10] Dreyer L. (2009). Latest Developments on SpaceX's Falcon 1 and Falcon 9 Launch Vehicles and Dragon Spacecraft, in IEEE Aerospace Conference (Big Sky, MT: IEEE).

[B11] Dubowsky S. Papadopoulos E. (1993). The kinematics, dynamics, and control of free-flying and free-floating space robotics systems. IEEE Trans. Robotics Autom. 9, 531–543.

[B12] Flores-Abad A. Ma O. Pham K. Ulrich S. (2014). A review of space robotics technologies for on-orbit servicing. Progr. Aerospace Sci. 68, 1–26. 10.1016/j.paerosci.2014.03.002

[B13] Goodman J. L. (2006). History of space shuttle rendezvous and proximity operations. J. Spacecr. Rockets 43, 944–959. 10.2514/1.19653

[B14] Hale W. Lane H. Lulla K. Chapline G. (2011). Wings in Orbit: Scientific and Engineering Legacies of the Space Shuttles 1971-2010. Washington, DC: NASA.

[B15] Ho J. Y. L. (1977). Direct Path method for flexible multibody spacecraft dynamics. J. Spacecr. Rockets 14, 102–110.

[B16] Hughes P. C. (1979). Dynamics of a chain of flexible bodies. J. Astronautical Sci. 27, 359–380.

[B17] Hughes P. C. (2004). Spacecraft Attitude Dynamics. Mineola, NY: Dover Publications.

[B18] Kennedy F. G. (2008). Orbital express: accomplishments and lessons learned, in Proceedings of the AAS Guidance and Control Conference (Breckenridge, CO: Univelt), 575–586.

[B19] Kuipers J. B. (2000). Quaternions and Rotation Sequences, in Proceedings of the International Conference on Geometry, Integrability and Quantization (Sofia: Coral Press Scientific Publishing), 127–143.

[B20] Liang B. Xu Y. Bergerman M. (1998). Mapping a space manipulator to a dynamically equivalent manipulator. J. Dyn. Syst. Meas. Control 120, 1–7.

[B21] Longman R. W. Lindberg R. E. Zedd M. F. (1987). Satellite-mounted manipulators - new kinematics and reaction moment compensation. Int. J. Rob. Res. 6, 87–103.

[B22] Marchesi M. (1997). Control strategy of a free-flying space manipulator, in Proceedings of the 8th International Conference on Advanced Robotics (ICAR '97) (Monterey, CA), 665–670

[B23] Moosavian S. A. A. Papadopoulos E. (2004). Explicit dynamics of space free-flyers with multiple manipulators via SPACEMAPLE. Adv. Robotics 18, 223–244. 10.1163/156855304322758033

[B24] Moosavian S. A.A. Papadopulos E. (2007). Free-flying robots in space: an overview of dynamics modeling, planning and control. Robotica 25, 537–547. 10.1017/S0263574707003438

[B25] Mukherjee R. Nakamura Y. (1992). Formulation of efficient computation of inverse dynamics of space robots. IEEE Trans. Robot. Autom. 8, 400–406.

[B26] Nakamura Y. Mukherjee R. (1989). Nonholonomic path planning of space robots, in Proceedings of 1989 International Conference on Robotics and Automation (Scottsdale, AZ: IEEE), 1050–1055.

[B27] Nanos K. Papadopoulos E. (2011). On the use of free-floating space robots in the presence of angular momentum. Intell. Serv. Robot. 4, 3–15. 10.1007/s11370-010-0083-2

[B28] Nenchev D. N. Yoshida K. (1999). Impact analysis and post-impact motion control issues of a free-floating space robot subject to a force impulse. IEEE Trans. Robot. Autom. 15, 548–557.

[B29] Nenchev D. N. Yoshida K. Vichitkulsawat P. Uchiyama M. (1999). Reaction null-space control of flexible structure mounted manipulator systems. IEEE Trans. Robot. Autom. 15, 1011–1023.

[B30] Oda M. (2000). Experiences and lessons learned from the ETS-VII robot satellite, in Proceedings of the 2000 IEEE International Conference on Robotics & Automation (San Francisco, CA: IEEE), 914–919.

[B31] Papadopoulos E. G. (1990). On the Dynamics and Control of Space Manipulators. Ph.D. Dissertation, Cambridge, MA: Massachusetts Institute of Technology.

[B32] Patten L. Evansa L. Oshinowo L. Ochisor M. Kazuharu N. Lodewijk A. (2002). International Space Station Robotics: A Comparative Study of ERA, JEMRMS and MSS, in 7th ESA Workshop on Advanced Space Technologies for Robotics and Automation ‘ASTRA 2002' (Noordwijk: ESA), 1–8.

[B33] Reed B. B. Smith R. C. Naasz B. Pellegrino J. Bacon C. (2016). The restore-L servicing mission, in AIAA Space Forum (Long Beach, CA: AIAA), 1–8.

[B34] Reintsema D. Thaeter J. Rathke A. Naumann W. Rank P. Sommer J. (2010). DEOS - the german robotics approach to secure and de-orbit malfunctioned satellites from low-earth orbits, in Proceedings of International Symposium on Artificial Intelligence, Robotics and Automation in Space (i-SAIRAS) (Sapporo,: JAXA), 244–251.

[B35] Roesler G. Jaffe P. Henshaw G. (2017). Orbital mechanics. IEEE Spectrum 54, 44–50. 10.1109/MSPEC.2017.7864756

[B36] Sallaberger C. (1997). Canadian space robotic activities. Acta Astronaut. 41, 239–246.

[B37] Sargent D. G. (1984). The Impact of Remote Manipulator Structurel Dynamics on Shuttle On-Orbit Flight Control, in 17th Fluid Dynamics, Plasma Dynamics, and Lasers Conference (Seattle, WA: AIAA).

[B38] Sato N. Wakabayashi Y. (2001). JEMRMS design features and topics from testing, in Proceeding of the 6th International Symposium on Artificial Intelligence and Robotics & Automation in Space: i-SAIRAS 2001 (St-Hubert, QC: CSA).

[B39] Shoemaker J. Wright M. (2004). Orbital express space operations architecture program, in Proceedings of SPIE 5088, Space Systems Technology and Operations (Orlando, FL), 56–65.

[B40] Siciliano B. Sciavicco L. Villani L. Oriolo G. (2010). Robotics. London: Springer-Verlag.

[B41] Stieber M. E. Trudel C. P. Hunter D. G. (1997). Robotic systems for the international space station, in Proceedings of the 1997 IEEE International Conference on Robotics and Automation (Albuquerque, NM: IEEE), 3068–3073.

[B42] Stoneking E. (2007). Newton-euler dynamic equations of motion for a multi-body spacecraft, in AIAA Guidance, Navigation and Control Conference and Exhibit, Guidance, Navigation, and Control and Co-located Conferences (Hilton Head, SC: AIAA), 1–13.

[B43] Ueda S. Kasai T. Uematsu H. (2010). HTV rendezvous technique and GN&C design evaluation based on 1st Flight on-orbit operation result, in AIAA/AAS Astrodynamics Specialist Conference, Guidance, Navigation, and Control and Co-located Conferences (Toronto, ON: AIAA), 1–12.

[B44] Umetani Y. Yoshida K. (1989). Resolved motion rate control of space manipulators with generalized Jacobian Matrix. IEEE Trans. Robotics Autom. 5, 303–314.

[B45] Vafa Z. Dubowsky S. (1987). On the dynamics of manipulators in space using the virtual manipulator approach, in IEEE International Conference on Robotics and Automation (Raleigh, NC).

[B46] Wei R. Jin M. H. Xia J. J. Xie Z. W. Shi J. X. Liu H. (2006). High fidelity distributed hardware-in-the-loop simulation for space robot, in Proceedings of the 2006 IEEE International Conference on Mechatronics and Automation (Luoyang: IEEE), 2150–2155.

[B47] Wertz J. R. (1978). Spacecraft Attitude Determination and Control. Boston, MA: Kluwer Academic Publishers.

[B48] Wie B. (2008). Space Vehicles Dynamics and Control 2nd Edn. Reston, VA: AIAA.

[B49] Xu W. Liu Y. Liang B. Xu Y. Li C. Qiang W. (2008). Non-holonomic path planning of a free-floating space robotic system using genetic algorithms. Adv. Robot. 22, 451–476. 10.1163/156855308X294680

[B50] Yoshida K. (2003). Engineering test satellite VII flight experiments for space robot dynamics and control: theories on laboratory test beds ten years ago, now in orbit. Int. J. Rob. Res. 22, 321–335. 10.1177/0278364903022005003

[B51] Yoshida K. Nakanishi H. (2003). Impedance matching in capturing a satellite by a space robot, in Proceedings of the 2003 IEEE/RSJ Intl. Conference on Intelligent Robots and Systems. (Las Vegas, NV: IEEE), 3059–3064.

[B52] Yoshida K. Nenchev D. N. (1998). A general formulation for under-actuated manipulators, Eighth International Symposium on Robotics Research (Shonan).

[B53] Yoshida K. Umetani Y. (1993). Control of space manipulators with generalized Jacobian, in Space Robotics: Dynamics and Control, eds Xu Y. Kanade T. (Boston, MA: Springer), 165–204.

